# An integrative assessment of *Dactylosoma* cf. *ranarum* (Apicomplexa: Dactylosomatidae) from *Pelophylax* water frogs

**DOI:** 10.1051/parasite/2026031

**Published:** 2026-06-09

**Authors:** Andrea Valigurová, Stanislav Hodeček, Veronika Michalková, Petr Papežík, Viktória Čabanová, Tatiana Kúdelová, Edward Charles Netherlands, Peter Mikulíček, Michal Benovics

**Affiliations:** 1 Department of Botany and Zoology, Faculty of Science, Masaryk University Kotlářská 2 611 37 Brno Czechia; 2 Department of Zoology, Faculty of Natural Sciences, Comenius University in Bratislava Ilkovičova 6 842 15 Bratislava Slovakia; 3 Institute of Zoology, Slovak Academy of Sciences Dúbravská cesta 9 845 06 Bratislava Slovakia; 4 Institute of Virology, Biomedical Research Center of the Slovak Academy of Sciences Dúbravská cesta 9 845 06 Bratislava Slovakia; 5 Department of Zoology and Entomology, University of the Free State, Bloemfontein Campus 9300 South Africa; 6 Unit for Environmental Sciences and Management, North-West University Potchefstroom 2520 South Africa

**Keywords:** Anura, Haemoparasites, Haemogregarine, Vector, Diptera, Slovakia

## Abstract

While various haemoparasites have been reported from anuran hosts, this study provides the first published record of dactylosomatid parasites (Dactylosomatidae Jakowska & Nigrelli, 1955 emend. Levine, 1971) in frogs from Slovakia. Of the 239 anurans screened for apicomplexans, 67 individuals belonging to three species of water frogs, *Pelophylax esculentus* (Linnaeus, 1758), *P. ridibundus* (Pallas, 1771), and *P. lessonae* (Camerano, 1882) were found to be infected with haemogregarines of the genus *Dactylosoma* Labbé, 1894. Our results demonstrate that the haemogregarine found in all three species of water frogs, collected from three different localities in western Slovakia, represents a single taxon that morphologically resembles the type species, *Dactylosoma ranarum* (Kruse, 1890). Based on comprehensive morphometric, morphological, and molecular data from newly collected isolates from *Pelophylax* frogs, including the type host, we provide an integrative assessment of *Dactylosoma* cf. *ranarum*. We also conducted molecular screening of dipterans collected from the study sites, which could serve as potential vectors of the parasite, but no representatives of *Dactylosoma* were detected in any of the examined specimens.

## Abstract (fr)

Bien que divers hémoparasites aient été signalés chez des hôtes anoures, cette étude fournit la première mention publiée de dactylosomatidés (Dactylosomatidae Jakowska & Nigrelli, 1955 emend. Levine, 1971) parasites de grenouilles de Slovaquie. Parmi les 239 anoures examinés pour la recherche d'apicomplexes, 67 individus appartenant à trois espèces de grenouilles, *Pelophylax esculentus* (Linnaeus, 1758), *P. ridibundus* (Pallas, 1771) et *P. lessonae* (Camerano, 1882) étaient infectés par des hémogrégarines du genre *Dactylosoma* Labbé, 1894. Nos résultats démontrent que l'hémogrégarine présente chez ces trois espèces de grenouilles, collectées dans trois localités différentes de l'ouest de la Slovaquie, représente un taxon unique qui ressemble morphologiquement, à *Dactylosoma ranarum* (Kruse, 1890). À partir de données morphométriques, morphologiques et moléculaires exhaustives issues d'isolats nouvellement collectés chez des grenouilles du genre *Pelophylax*, y compris l'hôte-type, nous proposons une évaluation intégrative de *Dactylosoma* cf. *ranarum*. Nous avons également effectué un criblage moléculaire des diptères collectés sur les sites d'étude, qui pourraient servir de vecteurs potentiels du parasite, mais aucun représentant de *Dactylosoma* n'a été détecté dans aucun des spécimens examinés.

## Introduction

Amphibians are often heavily infected by a wide range of pathogens due to their mixed terrestrial–aquatic habits, which expose them to different conditions that facilitate infection [26]. Despite this, they have historically received limited attention in parasitological research in Europe, and our current understanding of the diversity of their parasites remains inadequate. The primary focus of parasitological studies has traditionally been on parasites of significant epidemiological relevance, particularly those affecting mammals, including humans and domestic animals. To date, a total of six genera of apicomplexan haemoparasites (Apicomplexa Levine 1970, emend. Adl *et al.* 2012), belonging either to haemogregarines (Adeleorina) or haemococcidia (Eimeriorina), have been discovered in anuran hosts [63, 68]. Among them is the genus *Dactylosoma* Labbé, 1894 (Dactylosomatidae (Jakowska & Nigrelli, 1955) emend. Levine, 1971), for which only six species from anuran hosts have been described to date, and the complete life cycle of all remains unclear [21, 43, 56, 64, 100, 101]. Taxonomic disputes have surrounded *Dactylosoma* representatives over time. Most findings relied solely on morphological and morphometric data (*e.g*., [4, 6, 14, 43, 56, 60, 82, 105, 106]), which, given the morphological uniformity of the genus, may not guarantee completely reliable discrimination between individual *Dactylosoma* species.

According to Netherlands *et al.* [64], discrepancies began with the first described species, *Dactylosoma ranarum*, with Lankester [47] sometimes credited for this discovery (*e.g*., [4, 5, 51]). However, closer examination of the original descriptions [47, 48] revealed that he had been working with a species of *Lankesterella* Labbé, 1899 (initially known as *Drepanidium* erected by Lankester in 1882). The first description of *Dactylosoma ranarum* was provided by Kruse [43], who described the parasite from *Pelophylax* water frogs collected in the vicinity of Naples, Italy, originally naming it *Drepanidium ranarum* [105]. He proposed classifying the parasite among the haemogregarines and, should the generic name *Drepanidium* be rejected, naming it *Haemogregarina ranarum* (Kruse, 1890). Subsequently, some authors classified it as *H. ranarum* [13], while others considered it a malarial parasite [27]. It was not until Labbé [45] described the unique morphological features that distinguished this species from other proposed genera that it was formally established as a member of a newly erected genus *Dactylosoma*, based on: (1) different cell forms – cylindrical and amoeboid, sometimes pyriform; (2) areolar structure, vesicular nucleus, and hyaline cytoplasm; (3) absence of pigment and presence of retractile granules; (4) minimal effect on the host cell and its nucleus; and (5) merogony (“sporulation”) yielding 5–12 sporozoites arranged in a rosette- or fan-like pattern [45, 64]. While Labbé [45] originally named this species *Dactylosoma splendens*, it was later synonymised with *Dactylosoma ranarum* (Kruse, 1890) [64, 67, 105].

The life cycle of *Dactylosoma* spp. comprises two morphologically distinct merogonic cycles occurring within the peripheral blood of the vertebrate host. During primary merogony, large trophozoites and multinucleate meronts producing up to 20 merozoites through simultaneous peripheral budding can be observed [101]. This budding often occurs along one side of the meront, displaying a characteristic hand-like appearance from which the genus derives its name. The free merozoites then invade other erythrocytes, either repeating primary merogony or initiating secondary merogony, during which smaller secondary meronts generate up to eight merozoites. These may either recycle or transform into gamont stages, which are likely ingested by the vector during blood feeding and the development proceeds with gamogony and sporogony [4, 64]. However, the development of any *Dactylosoma* parasite in a natural vector has not yet been elucidated.

Although *D. ranarum* holds the distinction of being the first formally described intraerythrocytic apicomplexan parasite [5], knowledge of the genus *Dactylosoma* remains surprisingly limited. Therefore, in this study, we performed the microscopic and molecular screening for *Dactylosoma* representatives in western Slovakia, in order to identify the parasites to the species level, expand understanding of their host spectrum, and clarify aspects of their life cycle. Additionally, we conducted molecular screening of blood-sucking dipterans from the same areas, as these insects may serve as potential vectors of *Dactylosoma*. This study provides the first published record of blood apicomplexans in anurans from Slovakia and is among the few investigations of apicomplexan parasites in ectotherms in this country.

## Materials and methods

### Material collection

#### Ethics statement

Scientific permits for material collection and processing were provided by the Ministry of the Environment of the Slovak Republic (permit No. 519/2022-6.3).

#### Field sampling of anurans, blood collection, and processing

A total of 239 individuals representing nine anuran species from seven genera (Table 1) were collected from three localities in western Slovakia – Devín (48.1752, 16.9769), Rusovce (48.0564, 17.15400), and Moravský Svätý Ján (48.5760, 16.9535). Localities Devín and Rusovce were selected as they have been identified in our previous unpublished studies to harbour the hosts and parasites of interest. The sampling site in Rusovce was visited five times in total – in June and August 2022 (summer period), and in April (spring), June, and August 2023 (summer). Sampling in Devín was performed three times – in June 2022 (summer), and in April and May 2023 (spring) – as sampling in late summer was not possible due to the drying up of the water source. Additionally, Moravský Svätý Ján, was visited once in June 2024 (summer) based on our knowledge of the occurrence of the potential vector *Culex territans*, a mosquito species that primarily feeds on frog blood [11] and was absent from the previous two locations.

**Table 1:** Overview of collected frog species (Anura).

Family	Species	Locality	No. collected
Bombinatoridae	*Bombina bombina* (Linnaeus, 1761)	Bratislava – Devín	57
Bufonidae	*Bufo bufo* (Linnaeus, 1758)	Bratislava – Devín	2
	*Bufotes viridis* (Laurenti, 1768)	Bratislava – Devín	2
Hylidae	*Hyla arborea* (Linnaeus, 1758)	Bratislava – Devín	10
Pelobatidae	*Pelobates fuscus* (Laurenti, 1768)	Bratislava – Devín	3
Ranidae	*Pelophylax esculentus* (Linnaeus, 1758)	Bratislava – Devín, Bratislava – Rusovce, Moravský Svätý Ján	126
	*Pelophylax lessonae* (Camerano, 1882)	Bratislava – Rusovce, Moravský Svätý Ján	14
	*Pelophylax ridibundus* (Pallas, 1771)	Bratislava – Devín, Bratislava – Rusovce	24
	*Rana dalmatina* Fitzinger, 1838	Bratislava – Devín	1

The frogs were collected by hand at night and, after processing, were immediately released at the capture site. The species and sex (male/female) of the frogs were determined according to their morphological characteristics [29, 73, 90]. Subsequently, a small volume of blood was taken from each anuran via puncture of the facial vein using a sterile insulin syringe [24]. A portion of the blood samples collected at the Devín and Rusovce sites was used to prepare two to three thin blood smears per host specimen on clean glass slides. The remaining blood was placed into two sterile 1.5 mL microtubes; one with 96% ethanol for molecular analysis and the other 2.5% (v/v) glutaraldehyde in 0.1 M Sorensen’s phosphate buffer for transmission electron microscopy. Only molecular methods were used to examine the frog blood samples from Moravský Svätý Ján for the presence of *Dactylosoma*. Furthermore, sequence data of a *Dactylosoma* sp. isolated from a single *P. esculentus* collected as part of a separate survey (unpublished data) in Perpignan, France (42.690389, 2.726911) during May 2015 (spring) were included for phylogenetic comparison.

#### Field sampling and processing of potential *Dactylosoma* dipteran vectors

To search for potential insect vectors, we collected blood sucking dipterans using CDC style mosquito traps augmented with dry ice releasing CO_2_ set at the localities of Devín and Rusovce in May, July, and August 2023, and in Moravský Svätý Ján in June 2024. The traps were hung to a tree approximately 1.5 m above the ground at dusk and collected after dawn the next day. A total of 454 mosquitoes, representing three genera and seven species, and 746 blackflies from a single species were collected (Table 2). After collection, the dipterans were transported to the laboratory, where they were kept in a refrigerator at 4 °C for a few minutes to slow down their activity and subsequently identified to species based on their morphology [39, 42, 80]. Additionally, DNA extracted from pooled samples of 1,475 mosquitoes collected from various localities in western Slovakia, belonging to five genera and eleven species (Table 3; originally obtained for molecular detection of various microbial pathogens), was analysed for the presence of *Dactylosoma*.

**Table 2:** Overview of collected dipteran species (Diptera).

Family	Species	Locality	No. collected
Culicidae	*Aedes cantans* (Meigen, 1818)	Bratislava – Devín, Rusovce	43
	*Aedes cinereus* Meigen, 1818	Bratislava – Devín	10
	*Aedes sticticus* (Meigen, 1838)	Bratislava – Devín, Bratislava – Rusovce	78
	*Aedes vexans* (Meigen, 1830)	Bratislava – Devín, Bratislava – Rusovce	315
	*Anopheles maculipennis* (Meigen, 1818)	Bratislava – Devín, Bratislava – Rusovce	4
	*Culex territans* (Walker, 1856)	Moravský Svätý Ján	4
Simuliidae	*Simulium balcanicum* (Enderlein, 1924)	Bratislava – Devín, Bratislava – Rusovce	746

**Table 3:** Overview of mosquitos (Diptera: Culicidae) in pooled samples.

Species	Locality	No. collected
*Aedes caspius* (Pallas, 1771)	**Bratislava** – **Devín**	3
*Aedes cinereus/geminus* (Peus, 1971)	**Bratislava** – **Devín**, Suchohrad	4
*Aedes hungaricus* (Mihalyi, 1955)	**Moravský Svätý Ján**	4
*Aedes sticticus* (Meigen, 1838)	**Bratislava** – **Devín**, Draždiak, Devinske jazero, Zohor, Suchohrad	232
*Aedes vexans* (Meigen, 1830)	**Bratislava** – **Devín**, Bratislava – Draždiak, Devinske jazero, Gajary, Malé Leváre, Stupava, Suchohrad, Sekule	896
*Anopheles maculipennis* (Meigen, 1818)	Vysoká pri Morave, Gajary, Malé Leváre, Suchohrad, **Moravský svätý Ján**	61
*Anopheles plumbeus* (Stephens, 1828)	Zohor	1
*Coquillettidia richiardii* (Ficalbi, 1889)	Vysoká pri Morave, Gajary, Suchohrad, **Moravský Svätý Ján**	15
*Culex modestus* (Ficalbi 1889)	Vysoká pri Morave, Gajary, Malé Leváre, Suchohrad, Sekule, **Moravský Svätý Ján**	54
*Culex pipiens* (Linnaeus, 1758)	**Bratislava** – **Devín**, Bratislava – Draždiak, Bratislava – Pečniansky les, Vysoká pri Morave, Gajary, Suchohrad, **Moravský Svätý Ján**	136
*Culiseta annulata* (Schrank, 1776)	Vysoká pri Morave, Malé Leváre, **Moravský svätý Ján**	69

Note: Sites where mosquitoes were collected, coinciding with frog sampling locations for this study, are highlighted in bold.

### Light microscopic analysis

The prepared thin blood smears were fixed with absolute methanol and stained with a 5% Giemsa solution prepared in distilled water and adjusted to a pH of 7.2 [103]. Smear preparations were observed and photodocumented using a 100× immersion oil objective of an Olympus BX61 microscope equipped with DP71 digital camera.

Captured parasites were measured using the Stream Motion imaging software programme. Measurements were recorded in micrometres (μm), comprising the length and the width of the parasite, with the average, standard deviation, and range given. Parasitaemia (expressed as percentage of parasitised erythrocytes) was calculated after scanning 50 visual fields of approximately 20 erythrocytes, meaning approximately 10^3^ erythrocytes were examined per blood smear. Parasite prevalence was established as the proportion of infected individuals out of the total number of anurans sampled. Mean abundance of infection and mean intensity of infection were calculated from the parasitaemia values. Mean abundance of infection was determined by averaging parasitaemia across all sampled anurans, while the mean intensity of infection excluded data from uninfected ones. To compare the level of parasitaemia among individuals (*P. esculentus*, *n* = 33, 85% females) collected in April, June, and August 2023 in Rusovce, a Kruskal–Wallis test was used. Prior to the test, normality of data was tested via a Shapiro–Wilk test (*α* = 0.05). The analysis was performed in the R, v. 4.5.1 [74] using the “tidyr” [108], and “dplyr” [107] packages.

### Ultrastructural analysis

For transmission electron microscopy (TEM), the samples were fixed overnight in a freshly prepared 2.5% glutaraldehyde in 0.2 M Sorensen’s phosphate buffer (pH 7.2). Based on light microscopic analysis of blood smears, only samples with the highest parasitaemia were selected for further processing. The specimens were washed three times in the same buffer, post-fixed with 1% osmium tetroxide in the same buffer for 2 h at room temperature, dehydrated in a graded series of ethanol and embedded into Spurr resin (SPI-pon, SPI) blocks. The ultrathin sections were made using Ultracut UCT (Leica) microtome and stained according to the standard protocol [102]. Ultrathin sections were examined and photodocumented using a 1400 Flash (JEOL) transmission electron microscope.

### Molecular and phylogenetic analyses

#### Molecular detection of *Dactylosoma* in anuran blood

A small volume of blood from parasitised hosts preserved in 96% ethanol was transferred to a new microtube and residual ethanol was subsequently removed using a vacuum centrifuge at 60 °C. Afterwards, DNA was extracted from all the collected blood samples using a NucleoSpin^®^ Tissue kit (Macherey-Nagel, Düren, Germany), following the manufacturer’s standard protocol for human or animal tissue and cultured cells. After extraction, DNA was used for polymerase chain reaction (PCR) amplification undertaken in a Mastercycler^®^ EP Gradient Thermal Cycler (Eppendorf, Hamburg, Germany). The partial fragments (approximately 600 bp) of the 18S rDNA gene were amplified using the primer set HepF300 (5′–GTTTCTGACCTATCAGCTTTCGACG–3′) and HepR900 (5′–CAAATCTAAGAATTTCACCTCTGAC–3′) [99]. PCR reactions were carried out in volumes of 20 μL, containing 14 μL of FIREPol^®^ Master Mix (Solis BioDyne, Tartu, Estonia), 4 μL of RNase-Free Water, 0.5 μL of each primer (10 μM), and 1 μL of the extracted DNA. PCR conditions were as follows: initial denaturation at 95 °C for 15 min, followed by 35 cycles comprising denaturation at 95 °C for 45 s, annealing at 61 °C for 45 s, and extension at 72 °C for 1 min 30 s, followed by a final extension at 72 °C for 10 min. PCR products were subjected to gel electrophoresis at 100 V for 30 min on a 1.5% agarose gel stained with GoodView Nucleic Acid Gel Stain (SBS Genetech, Beijing, PR China) and then visualised under ultraviolet light using an UV transilluminator. Finally, the products were purified by adding 2 μL of EPPiC Fast (A&A Biotechnology, Gdańsk, Poland) to 10 μL of PCR product, according to the manufacturer’s protocol and sent to a commercial sequencing company (Macrogen Europe, Amsterdam, Netherlands) for sequencing in both forward and reverse directions.

The obtained sequence chromatograms were assembled and edited to produce a partial 18S rDNA consensus sequence using the bioinformatics software Geneious Prime [37]. Species identity was verified against previously published sequences using the Basic Local Alignment Search Tool (BLAST) (https://blast.ncbi.nlm.nih.gov/Blast.cgi). Subsequently, all available sequences for *Dactylosoma* spp. and representative sequences of four other genera parasitising in blood of amphibians and reptiles (i.e., *Haemogregarina*, *Hepatozoon*, *Hemolivia*, and *Karyolysus*) were downloaded from the NCBI GenBank, along with the ortholog sequences of *Klossia helicina* [GenBank accession number HQ224955] and *Adelina dimidiata* [DQ096835], which were selected as the outgroup [64]. The newly obtained sequences were aligned with all downloaded sequences using the MAFFT 7 online alignment tool (https://mafft.cbrc.jp/alignment/server/index.html) [36]. The bioinformatics software MEGA11 [95] was used to manually trim the alignment and remove any alignment gaps and ambiguities. Phylogenetic reconstructions were conducted through Bayesian inference (BI) using MrBayes 3.2.7 and Maximum likelihood (ML) analysis using RAxML 8.1.12 [81, 91]. For both analyses, the generalised time reversible (GTR) [97] nucleotide substitution model was applied. In the BI analysis, the Markov Chain Monte Carlo (MCMC) algorithm was run for 10^6^ generations, sampling every 100 generations. The initial 30% of the trees were discarded as “burn-in”. In the ML analysis, nodal support was evaluated based on 1,000 bootstrap replicates. The resulting phylogenetic trees were visualised and summarised into a consensus tree using FigTree 1.4.4 [77].

#### Molecular detection of *Dactylosoma* in dipterans

The mosquitos and blackflies were separated into 1.5 mL microtubes according to the species identification and locality, with each tube containing ~20 individuals (or fewer in the case of a small number of collected individuals). Homogenisation of each sample was performed with a motorised pestle mixer. DNA extraction, PCR amplification, and gel electrophoresis were carried out in the same way as for frog blood processing.

## Results

### Prevalence of *Dactylosoma* cf. *ranarum* in frogs from western Slovakia

From the 239 anurans screened, a total of 67 individuals (28.03%) belonging to three species (*P. esculentus*, *P. ridibundus*, and *P. lessonae*) were found to be infected with *Dactylosoma*. The prevalence in all frogs collected in Devín was 12.12% (12/99), in Rusovce 37.29% (44/118), and Moravský Svätý Ján 45.45% (10/22) (Table 4). The overall prevalence of *Dactylosoma* in the three infected species was 42.86% (54/126) in *P. esculentus*, 45.83% (11/24) in *P. ridibundus*, and 14.29% (2/14) in *P. lessonae*.

**Table 4:** Prevalence of *Dactylosoma* cf. *ranarum* SK at the sampling localities in Slovakia.

Locality	Host species	No. of collected anurans	No. of infected anurans	Parasite prevalence (%)
Devín	*Bombina bombina*	57	0	0
	*Bufo bufo*	2	0	0
	*Bufotes viridis*	2	0	0
	*Hyla arborea*	10	0	0
	*Pelobates fuscus*	3	0	0
	***Pelophylax esculentus***	**1**	**1**	**100**
	***Pelophylax ridibundus***	**23**	**11**	**47.83**
	*Rana dalmatina*	1	0	0
Rusovce	***Pelophylax esculentus***	**114**	**44**	**38.60**
	*Pelophylax lessonae*	3	0	0
	***Pelophylax ridibundus***	**1**	**0**	**0**
Moravský Svätý Ján	***Pelophylax esculentus***	**11**	**9**	**81.82**
	***Pelophylax lessonae***	**11**	**2**	**18.19**
**Total**		**239**	**67**	**28.03**

Note: Samples positive for *Dactylosoma* cf. *ranarum* SK are highlighted in bold.

Additional infection parameters were assessed by light microscopy of blood smears and, therefore, are available only for water frogs collected at the Devín and Rusovce sites. The mean intensity of infection (in %) was 1.7 ± 2.0 (0.1–8.7) in *P. esculentus* (*n* = 45) and 2.3 ± 2.0 (0.4–6.4) in *P. ridibundus* (*n* = 11); that is, on average, 17 and 23 out of 1,000 erythrocytes observed in an infected frog were parasitised by *Dactylosoma*, respectively. The mean abundance of infection (in %) was 0.7 ± 1.5 (0–8.7) in *P. esculentus* and 1.1 ± 1.8 (0–6.4) in *P. ridibundus*, i.e. on average, 7 and 11 out of 1,000 erythrocytes observed in a frog, regardless of its infection status, were parasitised by *Dactylosoma*, respectively.

The Kruskal–Wallis test showed no significant effect of the sampling month on the parasitaemia of *Dactylosoma* in *P. esculentus* (χ^2^ = 3.0244, *p* = 0.2204). However, the analysis of the seasonal dynamics of other epidemiological parameters showed a relatively clear and mutual trend. In April (spring), the prevalence (32.26%), intensity (1.96%), and abundance (0.63%) were moderate. In June (summer period), all three values increased significantly, with prevalence at 45.16%, intensity at 2.45%, and abundance at 1.11%. In August (summer), the values for all parameters fell to the lowest figures, with prevalence at 30%, intensity at 0.8%, and abundance at 0.24%.

### Supplementary description of *Dactylosoma ranarum* (Kruse, 1890) from specimens collected in this study

Phylum: Apicomplexa Levine, 1970

Class: Conoidasida Levine, 1988

Subclass: Coccidia Leuckart, 1879

Order: Eucoccidiorida Léger, 1911

Suborder: Adeleorina Léger, 1911

Family: Dactylosomatidae (Jakowska & Nigrelli, 1955 emend. Levine, 1971)

Genus: *Dactylosoma* Labbé, 1894

Type species: *Dactylosoma ranarum* (Kruse, 1890)

#### *Dactylosoma* cf. *ranarum* (Kruse, 1890)

**Type host:** *Pelophylax esculentus* (Linnaeus, 1758)

**Other hosts:** *Amnirana albolabris* (Hallowell, 1856), *Amnirana galamensis* (Dumeril and Bibron, 1841), *Fejervarya limnocharis* (Gravenhorst, 1829), *Hylarana guentheri* (Boulenger, 1882), *Hyperolius marmoratus* (syn. *Rappia marmorata*), *Pelophylax ridibundus* (Pallas, 1771), *Pelophylax nigromaculatus* (Hallowell, 1861), *Pelophylax saharicus* (Boulenger in Hartert, 1913), *Pyxicephalus adspersus* Tschudi, 1838, *Ptychadena oxyrhynchus* (Smith, 1849), *Ptychadena submascareniensis* (Guibe and Lamotte, 1953), *Rhinella marina* (Linnaeus, 1758), *Sclerophrys regularis* (Reuss, 1833), *Sylvirana guentheri* (Boulenger, 1882) (for review, see [4, 64, 105], and this study). *Note:* This list includes records derived in part from older literature; some identifications may therefore be inaccurate and should be interpreted with caution.

It should also be noted that in some cases, the hosts were more likely infected with several different blood parasites [64].

**Hosts in this study:** *Pelophylax esculentus* (Linnaeus, 1758), *Pelophylax lessonae* (Camerano, 1882), *Pelophylax ridibundus* (Pallas, 1771).

**Site of infection:** Peripheral blood.

**Definitive host and vector:** Unknown.

**Type locality:** An exact locality was not provided in the original description, only reference to frogs being collected in the vicinity of Naples, Italy.

**Other localities:** Cosmopolitan, reported across Europe, Central and South America, Africa, and Asia (for review, see [4, 64, 105], and this study).

**Locality in this study:** Slovakia: Bratislava – Devín (48.1752, 16.9769), Bratislava – Rusovce (48.0564, 17.15400), and Moravský Svätý Ján (48.5760, 16.9535), all in western Slovakia.

**Materials deposited:** The voucher material (DR1/2022, DR2/2022, DR3/2022) consists of Giemsa-stained blood smears containing representative developmental stages of *D.* cf. *ranarum.* The slides were deposited at the Department of Zoology, Faculty of Natural Sciences, Comenius University in Bratislava, Slovakia, where they are accessible for future studies. Additionally, a DNA sample collected from the same specimen as slide DR1/2022 (SK-API1exA) was deposited in the same collection as the voucher material.

**Representative DNA sequence:** A nucleotide sequence of the partial gene for 18S rDNA (629 bp, GenBank accession number PZ099871). No intraspecific variability was found in the studied material.

The developmental stages of *D. ranarum* observed in the Giemsa-stained blood smears include (i) trophozoites, (ii) young primary meronts, (iii) primary meronts, (iv) primary merozoites, (v) young secondary meronts, (vi) secondary meronts, (vii) secondary merozoites, and (viii) gamonts. Most of these stages were observed within erythrocytes, although some trophozoites and gamonts were also occasionally detected within leukocytes. Typically, intracellular stages, including trophozoites, meronts, and immature gamonts, were also observed extracellularly, primarily near destroyed erythrocytes.

**Figure 1:**
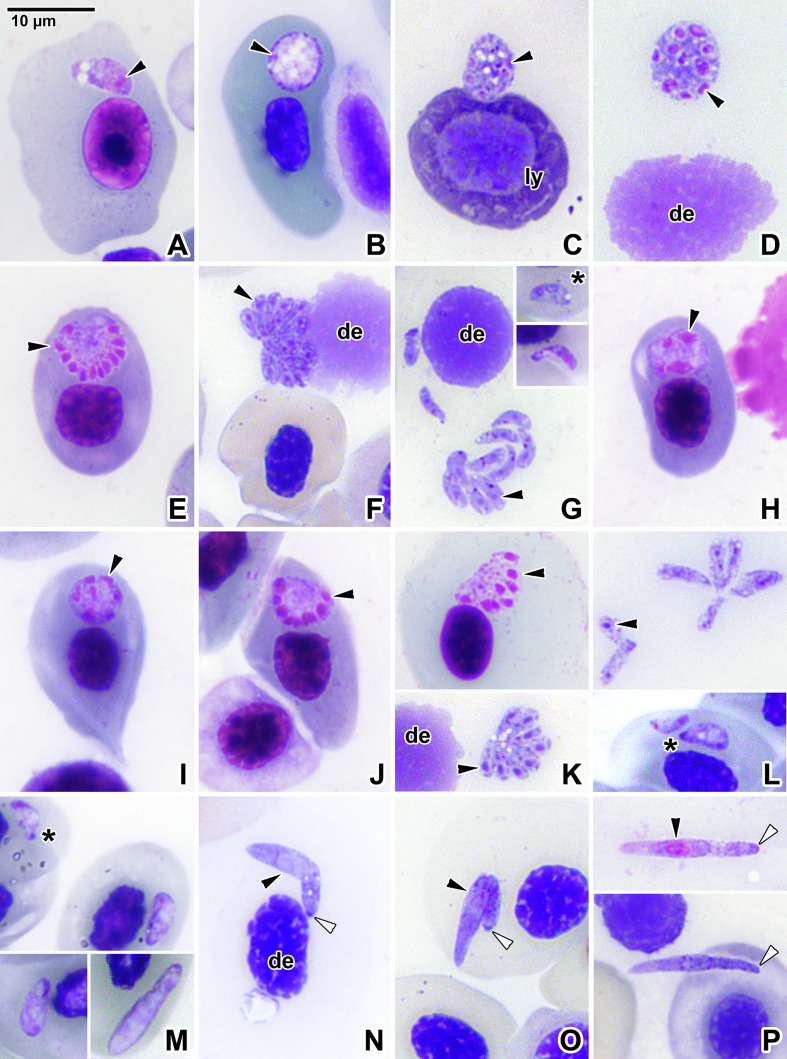
*Dactylosoma* cf. *ranarum* SK in blood smears of *Pelophylax esculentus*. A) Trophozoite. B–G) Primary merogony: B) Early meront. C; D) Young primary meront released from host cell, presumably due to cell damage or lysis. The meront in C appears to be partially protruding into the lymphocyte, suggesting a possible interaction. E; F) Primary meront with 12 budding merozoites. G) Primary merozoites. The top inset shows one of the merozoites that has already invaded the nearest erythrocyte; the bottom inset shows another primary merozoite from a different smear preparation. H–L) Secondary merogony: H; I) Young secondary meront. J) Maturing secondary meront with eight nuclei. K) Secondary meronts with eight budding merozoites. L) Secondary merozoites. M) Immature gamonts at various stages of development and a secondary merozoite. N) Immature gamont released from degraded erythrocyte. O) Mature intracellular gamont. P) Mature extracellular gamonts. Bright-field microscopy, Giemsa staining. Scale bar applies to all micrographs. Black a*rrowhead* – condensed chromatin/nucleus, *black asterisk –* merozoite following erythrocyte invasion, *de* – degrading erythrocyte, *ly* – lymphocyte, *white arrowhead* – apical end of gamont.

**Figure 2:**
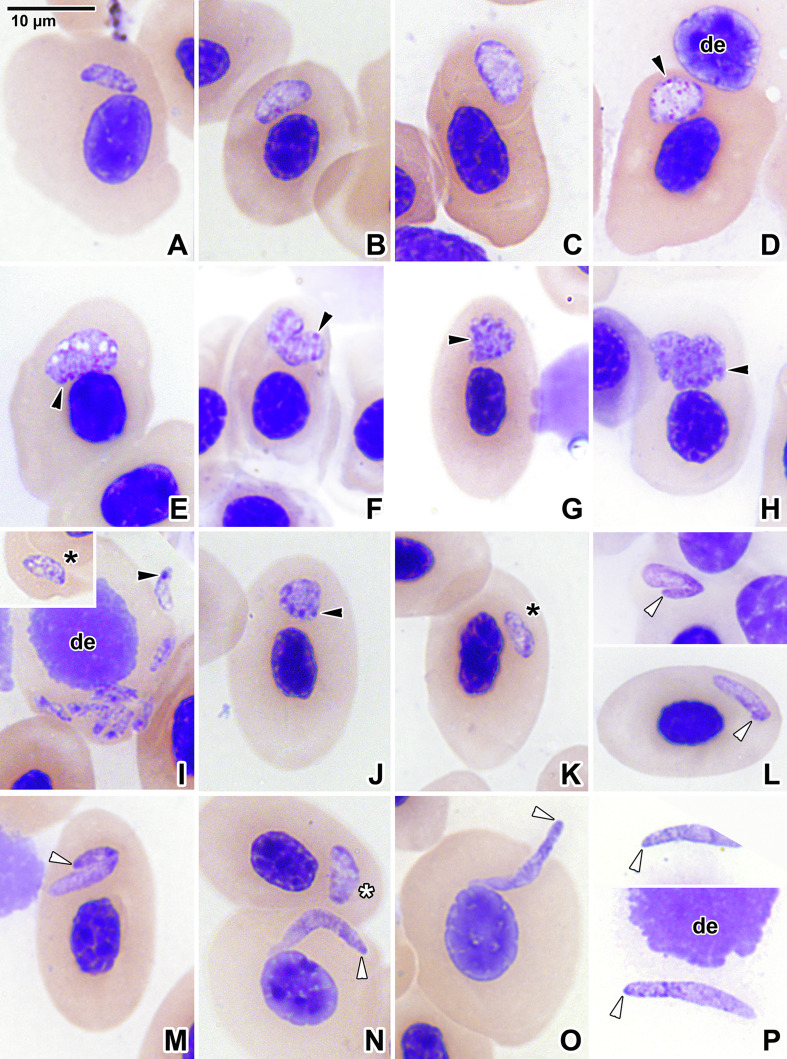
*Dactylosoma* cf. *ranarum* SK in blood smears of *Pelophylax ridibundus.* A) Trophozoite. B; C) Trophozoite transforming into meront stage. D–I) Primary merogony: D; E) Early primary meront. F) Young primary meront. G) Maturing primary meront with initial merozoite formation. H) Primary meront with budding merozoites. I) Primary merozoites. The inset shows a primary merozoite following erythrocyte invasion. J–K) Secondary merogony: J) Maturing secondary meront with five clearly visible nuclei. K) Secondary merozoite following erythrocyte invasion. L) Immature intracellular gamonts at different stages of development. M) Mature intracellular gamont. N) Mature intracellular gamont straightening prior to exiting the erythrocyte. O) Mature gamont emerging from an erythrocyte. P) Extracellular immature (top) and mature (bottom) gamont released from damaged erythrocytes. Bright-field microscopy, Giemsa staining. Scale bar applies to all micrographs. *Black arrowhead* – condensed chromatin/nucleus, *black asterisk –* merozoite after invasion of erythrocyte, *de* – degrading erythrocyte, *white arrowhead* – apical end of gamont; *white asterisk –* trophozoite.

**Trophozoites** (Figs. 1A, 2A–2C): Elongated to ovoid (depending on maturation stage) in shape, typically tapering towards one end, measuring 5.76 ± 0.64 (4.24–6.79) μm in length and 2.68 ± 0.39 (1.82–3.97) μm in width (*n* = 50). Small, round and dense nucleus located in the middle or towards the rounded end with chromatin staining pinkish or purplish. Cytoplasm staining whitish purple or pink-purple to purple, with vacuoles and non-staining inclusions observed in some cases, usually near the blunt end. No significant distortion of parasitised erythrocytes or displacement of their nucleus detected.

**Young primary meronts** (Figs. 1B–1C, 2D–2E): Roundish in shape, measuring 7.36 ± 0.51 (6.51–8.40) μm in length and 3.52 ± 0.55 (2.65–4.94) μm in width (*n* = 30). Multinucleate with nuclear division occurring close to the pellicle, chromatin staining purple or dark pink. Cytoplasm staining whitish purple or pink-purple to purple and packed with vacuoles or inclusions of various size, sometimes occupying up to half of the cell. Slight displacement of the host cell nucleus and distortion of the host cell occasionally present.

**Primary meronts** (Figs. 1E–1F, 2F–2H): Varying in shape, usually between round, amoeboid, and dactylate, measuring 7.41 ± 0.61 (6.17–8.49) μm in length and 6.39 ± 0.68 (5.20–7.81) μm in width (*n* = 20). Multinucleate and showing up to 16 nuclei (mostly 12) located peripherally with chromatin staining dark pink or dark purple. Cytoplasm staining bluish purple, purple or pink-purple, rarely with small cytoplasmic vacuoles or non-staining inclusions. Host cells occasionally showing displacement of their nucleus and a slight distortion.

**Primary merozoites** (Figs. 1G, 2I): Pear-shaped or crescent-shaped, measuring 3.71 ± 1.05 (2.05–5.52) μm in length and 1.18 ± 0.26 (0.64–1.76) μm in width (*n* = 50). Small stages grouping together in erythrocytes or scattered freely between blood cells, usually in numbers up to 16 (most often 12). Occasionally, solitary intraerythrocytic merozoites are observed after erythrocyte invasion. Roughly spherical nucleus mostly located towards one end of merozoite, staining light to dark purple or dark pink. Cytoplasm containing small vacuoles and staining light purple or pink-purple to bluish-purple. No significant host cell distortion or displacement of host cell nucleus documented.

**Young secondary meronts** (Figs. 1H–1I): Roundish to round, measuring 4.91 ± 0.40 (4.25–5.33) μm in length and 4.17 ± 0.39 (3.54–4.78) μm in width (*n* = 7). Only rarely detected in blood smears. Multinucleate, showing up to eight nuclei located close to their pellicle with chromatin staining dark pink or dark purple. Cytoplasm staining whitish purple to purple and rarely containing small vacuoles. Slight displacement of the host cell nucleus and host cell distortion occasional detected.

**Secondary meronts** (Figs. 1J–1K, 2J): Dactylate or fan-like in appearance, measuring 6.24 ± 0.65 (5.26–7.75) μm in length and 5.29 ± 0.28 (4.83–5.88) μm in width (*n* = 30). Multinucleate, showing up to eight nuclei located peripherally on one side of the cell with chromatin staining dark pink or dark purple. Cytoplasm staining whitish-purple, pink-purple to bluish-purple, rarely containing small vacuoles. Occasionally causing slight displacement of the host cell nucleus and host cell distortion.

**Secondary merozoites** (Figs. 1L, 1M-top micrograph, 2K): Crescent or pear-shaped, measuring 3.52 ± 0.57 (2.24–4.42) μm in length and 1.11 ± 0.25 (0.71–1.66) μm in width (*n* = 23). Small and relatively rare stages, observed in clusters in erythrocytes or scattered freely between blood cells, in numbers up to eight. Solitary intraerythrocytic merozoites present after erythrocyte invasion. Spherical, slightly elongated, or bean-shaped nucleus mostly positioned toward one end of the cell, staining dark purple to dark pink. Cytoplasm staining whitish- to bluish-purple or pink-purple and containing larger and more numerous vacuoles compared to primary merozoites. No significant host cell distortion or displacement of host cell nuclei observed.

**Immature gamonts** (Figs. 1M–1N, 2L, 2P–top micrograph): Cylindrical and slightly tapering to one end to elongated depending on stage of development, measuring 8.56 ± 2.21 (5.05–11.87) μm in length and 2.19 ± 0.33 (1.38–2.75) μm in width (*n* = 33). Straight or slightly flexed, with increasing dimensions, more often recurved within the host erythrocyte. The cytoplasm pale, staining faintly pink-purple, with a few dark pink inclusions and an indistinct nucleus. Observed mostly intracellularly in erythrocytes. No significant host cell distortion or displacement of the host cell nucleus detected.

**Mature gamonts** (Figs. 1O–1P, 2M–2O, 2P–bottom micrograph): Elongated and vermicular, measuring 13.43 ± 1.20 (11.12–15.94) μm in length and 1.69 ± 0.37 (1.12–2.71) μm in width (*n* = 50). Straight or slightly flexed in the middle, usually slightly tapering at both ends. Large round to oval nucleus located in or near the centre of the cell, with peripheral chromatin and karyosome (only rarely distinct) staining light purple or dark pink. The cytoplasm staining whitish- to bluish purple or pink-purple, only rarely containing small vacuoles. Observed extracellularly, intracellularly in erythrocytes, or potentially invading leukocytes. Intracellular gamonts were mostly recurved within the host erythrocyte, folding at or just posterior to the region of their nucleus. No significant host cell distortion or displacement of the host cell nucleus detected.

### Remarks and differential diagnosis

Since the original descriptions of *D. ranarum* are based solely on morphological and morphometric observations supported by drawings [43, 67, 105], and do not include any molecular data, the taxonomic status of this species remains problematic. The studied species of *Dactylosoma*, found in Slovakia (SK) and hereinafter referred to as *D.* cf. *ranarum* SK, shares phenotypic characteristics typical of the genus and closely resembles *D. ranarum*. Although *D.* cf. *ranarum* SK generally falls within the morphological descriptions of *D. ranarum* (Kruse, 1890) and *Dactylosoma* sp. from *P. lessonae* provided by Netherlands *et al.* [64], such as round shape of early meronts, dactylate appearance of mature meronts, and elongated and slender gamonts, it significantly deviates in morphometric characteristics from both these species (Table 5). However, the original description of *D. ranarum* by Kruse [43] appears to be somewhat broad and does not align well with the reported sizes of other *Dactylosoma* species. Compared to the *Dactylosoma* sp. described by Netherlands *et al.* [64], the parasite observed in our study showed significantly smaller dimensions at all developmental stages. The greatest morphometric differences were observed in primary meronts and gamonts. It is unclear whether this discrepancy could be due to the limited sample size in previous studies, potentially limiting the capturing of greater variation in cell dimensions, or possible artifacts that may arise during processing of blood smears (i.e. large blood components may be pushed to the edges of the slide, leading to peripheral flattening of other blood components, which then appear larger under the light microscope). This confirms the conclusion of other studies that although morphometric characteristics provide valuable insights, typical morphological features should be considered more important for the identification of haemoparasites [15, 110]. However, even in this respect *D.* cf. *ranarum* SK differs from the species mentioned above since we observed a different number of nuclear divisions and resulting merozoites during both primary and secondary intraerythrocytic merogony. In contrast to this study documenting production of up to 16 primary (but mostly 12) merozoites and 8 secondary merozoites, previous studies on *D. ranarum* described the large primary meronts producing from 4 to 16 merozoites and smaller secondary meronts producing 6 merozoites [4, 5, 12, 56, 67, 96].

**Table 5:** Overview of morphometric data on developmental stages of anuran *Dactylosoma* spp.

Species	Host(s)	Country	Morphometric data (μm)				Reference
			Trophozoites	Meronts	Merozoites	Gamonts	
*Dactylosoma* cf. *ranarum* SK	*Pelophylax esculentus*, *Pelophylax ridibundus*	Slovakia	5.76 × 2.78	YP: 7.36 × 3.52, P: 7.41 × 6.39	P: 3.71 × 1.18	13.43 × 1.69	Current study
	*Pelophylax lessonae*			YS: 4.91 × 4.17, S: 6.24 × 5.29	S: 3.52 × 1.11		
*Dactylosoma* sp.	*Pelophylax lessonae*	Belgium	6.3 × 3.8	YP: 9.4 × 8.0, P: 11.3 × 9.1	P: 4.0 × 1.9	9.8 × 2.3	[64]
				YS: 6.2 × 4.5, S: 7.7 × 6.6	S: 4.1 × 2.3		
*D. ranarum*	*Pelophylax esculentus*	Italy	3.0–4.0 × 1.5–2.0	P: 10.0–15.0 × 2.0–3.0	P: 2.8 × 0.7,	5.0–8.0 × 1.5–3.0	[43]
				S: 9.0 × 4.0	S: 2.0–3.0 × 1.0–1.5		
*D. ranarum*	*Pelophylax esculentus*	France (Corsica)	N/A	P: 7.3 × 7.3	P: 4.3 × 1.3	7.0 × 3.4	[6]
				S: 4.7 × 4.7	S: 3.4 × 0.9		
*D. ranarum*	*Fejervarya limnocharis*	China	N/A	P: 7.87 × 7.87	N/A	7.66 × N/A	[106]
*D. ranarum*	*Pelophylax nigromaculatus*	China	N/A	P: 6.01 × 6.01	N/A	6.5 × N/A	[106]
*D*. *amphibia*	*Leptodactylus latrans*, *Rhinella diptycha*, *Trachycephalus typhonius*	Brazil	5.37 × 2.99	YP: 6.78 × 3.95, P: 10.10 × 7.37	P: 3.05 × 1.72	N/A	[101]
				YS: 6.13 × 4.09, S: 7.01 × 3.99	S: 5.33 × 4.39		
*D*. *kermiti*	*Ptychadena anchietae*, *Sclerophrys gutturalis*	South Africa	6.7 × 3.5	YP: 7.8 × 5.7, P: 9.9 × 6.9	P: 5.6 × 2.7	10.6 × 2.2	[64]
				YS: 5.4 × 4.6, S: 7.2 × 5.7	S: 4.8 × 2.3		
*D*. *piperis*	*Leptodactylus labyrinthicus*	Brazil	7.40 × 3.75	YP: 5.20 × 5.53, P: 8.59 × 6.73	P: 7.45 × 2.90	N/A	[100]
				YS: 6.10 × 4.15, S: 6.95 × 4.89	S: 6.20 × 1.50		
*D*. *sylvatica*	*Lithobates sylvaticus*	Canada	7.0–8.5 × 6.3–7.6	P: 7.4–11.5 × 7.0–9.3	S: 4.4–5.9 × 1.1–2.0	7.0–12.6 × 1.5–3.0	[21]
				S: 5.2 × 4.0			
*D*. *taiwanensis*	*Fejervarya limnocharis*	Taiwan	7.3 × 3.9*	S: 6.9–7.9 × 5.6–7.3*	N/A	11.8–13.6 × 2.1–2.9*	[56]
*Dactylosoma* sp.	*Rhinella major*, *Rhinella marina*	Brazil	6.5–8.2 × 2.5–7.0	P: 6.5–5.4 × 8.4–6.8	S: 3.2–2.7 × 1.9–1.3	13.8–16.0 × 2.1–2.3	[14]
				S: 4.2–4.0 × 3.6–3.2			
*Dactylosoma* sp.	*Rhinella marina*	Costa Rica	N/A	P/S: 4–7 × 4–7	N/A	7 1–10 × 2–4	[82]

YP – young primary, P – primary, YS – young secondary, S – secondary, N/A – not available. *Measurements obtained from figure scale bar by Netherlands *et al.* [64].

Netherlands *et al.* [64] reported up to 12 merozoites during primary merogony and up to 6 merozoites during secondary merogony of *Dactylosoma* sp. from *P. lessonae* and speculated that it rather corresponds to description of *D. splendens* (synonymised with *D. ranarum*). It is unclear whether this difference could also be due to the limited sample size in their study (*i.e.* description on a sample from only a single infected individual). Furthermore, *D. splendens* was synonymised with *D. ranarum* more than 100 years ago [67, 105], at a time when the close resemblance between different species was not well understood [64]. Since precise morphometric data are not available from the original description, we cannot confirm that *D. splendens* indeed represents a synonym of *D. ranarum.* Moreover, the molecular data on these species are limited to two available sequences of *D. ranarum* [9], which were provided without accompanying morphological identification of the parasite.

In comparison to the less closely related *Dactylosoma* species, some developmental stages of *D.* cf. *ranarum* SK exhibit similar morphometric values to those of *D. kermiti*, *D. piperis*, and *D. amphibia* [64, 100, 101]. However, morphologically, significant differences can be observed between *D.* cf. *ranarum* SK and the above-mentioned species. All three species differ from *D.* cf. *ranarum* SK in the number of merozoites produced during primary merogony (up to 14 in *D. kermiti*, up to 10 in *D. piperis*, and up to 20 in *D. amphibia*). Furthermore, only the number of secondary merozoites in *D. piperis* matches that of *D.* cf. *ranarum* SK, as both *D. kermiti* and *D. amphibia* produce only up to 6 secondary merozoites. In addition, *D. piperis* can be clearly distinguished from *D.* cf. *ranarum* SK by its unique elongated trophozoites. When comparing *D.* cf. *ranarum* SK with *D. taiwanensis* and *D. sylvatica*, mostly different morphometric values were observed. Although the exact number of primary merozoites was not specified for these species, they both produce up to 8 merozoites during secondary merogony [21, 56], matching the number of secondary merozoites of *D.* cf. *ranarum* SK.

### Subcellular organisation

Intraerythrocytic stages, including trophozoites, and primary and secondary meronts with budding merozoites, and gamonts were detected in ultrathin sections of anuran blood samples. All *Dactylosoma* stages observed were contained within a parasitophorous vacuole with numerous membranous extensions into the host cell cytoplasm.

**Figure 3:**
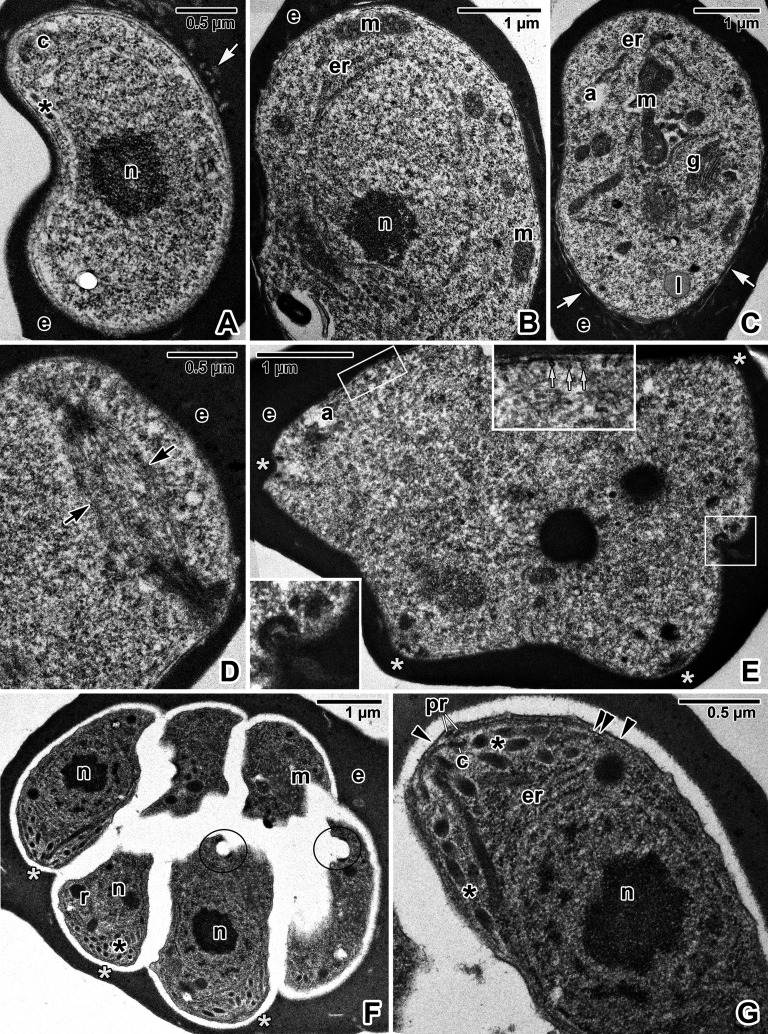
Subcellular organisation of *Dactylosoma* cf. *ranarum* SK trophozoites and primary merogonic stages. A) Trophozoite showing remnants of the apical complex. B; C) Trophozoite transforming into a meront stage. D) Early meront displaying the mitotic spindle. E) Young meront showing the initial stages of merozoite budding. F) Primary meront with budding merozoites. G) Anterior region of the merozoite shown in F. Transmission electron microscopy. *a* – amylopectin granule(s), *black arrowhead* – parasite plasma membrane, *black arrows* – mitotic spindle, *black asterisk* – microneme(s), *black circle* – micropore, *c* – conoid, *double black arrowhead* – inner membrane complex, *e* – erythrocyte, *er* – rough endoplasmic reticulum, *g* – Golgi apparatus, *l* – lipid droplet, *m* – mitochondrion, *n* – nucleus, *pr* – polar rings, *small white arrow* – subpellicular microtubule*, white arrow* – parasitophorous vacuole, *white asterisk* – apical pole of a budding merozoite.

**Trophozoites** (Figs. 3A–3C) distinguished by their ovoid or irregularly oval shape, usually somewhat flexed. They were covered by a three-layered pellicle, composed of the plasma membrane and the closely apposed inner membrane complex. In early stages, remnants of the apical complex were still present (Fig. 3A). The cytoplasm of mature trophozoites exhibited medium electron density and contained prominent mitochondria with tubular cristae, a well-developed rough endoplasmic reticulum, Golgi apparatus, a few amylopectin granules, and large lipid droplets (Figs. 3B–3C). The large nucleus, with a prominent nucleolus, was situated at the centre of the cell.

**Young meronts** (Figs. 3D–3E) were observed only rarely in ultrathin sections. The earliest stage corresponded to the onset of trophozoite transformation into a meront, as evidenced by the presence of a mitotic spindle (Fig. 3D). The subsequent stage was an early meront with an amoeboid shape and visible apical protrusions of emerging merozoites, including a clearly distinguishable conoid (Fig. 3E). The cytoplasm exhibited electron density similar to that of mature trophozoites. At this stage, a prominent micropore showing signs of feeding activity was also observed between the budding merozoites.

**Primary meronts** (Figs. 3F–3G) exhibited an irregular shape with peripherally budding merozoites. A maximum of six budding merozoites were visible per ultrathin section, and they appeared to develop synchronously. The budding merozoites possessed a well-developed three-layered apicomplexan pellicle and contained all organelles of the apical complex, including a conoid, a pair of preconoidal rings, two polar rings and associated subpellicular microtubules, micronemes, and a rhoptry with a crystalline appearance (Fig. 3G). A large micropore was observed in the anterior region of developing merozoites (Fig. 3F). The cytoplasm of primary merozoites was denser than in previous stages and contained a spherical nucleus with a large nucleolus, rough endoplasmic reticulum, a large mitochondrion, and a few small lucent vacuoles. No amylopectin granules were observed in the cytoplasm of primary merozoites.

**Figure 4:**
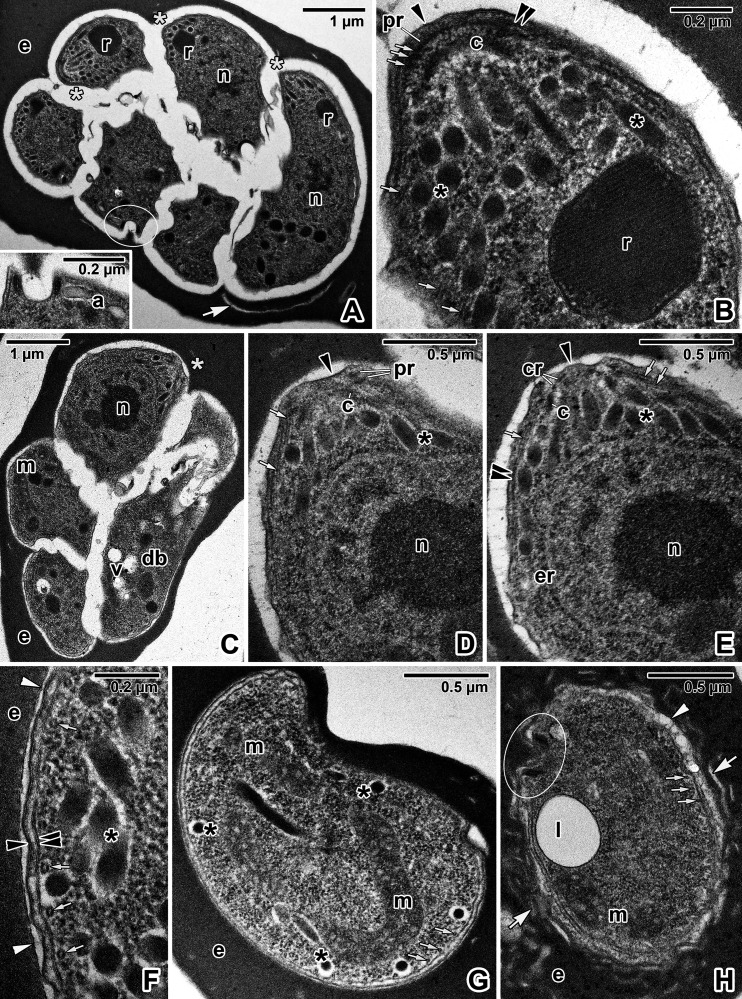
Subcellular organisation of *Dactylosoma* cf. *ranarum* SK secondary merogonic stages. A) Meront with budding merozoites. B) Apical end of the merozoite shown in A. C) Meront with budding merozoites. D; E) Anterior region of the merozoite shown in C (two different sections). F) High magnification of the pellicle and subpellicular microtubules in a budding merozoite. G) Tangential section through the anterior region of a merozoite following erythrocyte invasion. H) Cross-sectioned posterior region of a merozoite with a prominent micropore after erythrocyte invasion. Transmission electron microscopy. *a* – amylopectin granule, *black arrowhead* – parasite plasma membrane, *black asterisk* – microneme(s), *c* – conoid, c*r* – preconoidal rings, *db* – dense body, *double black arrowhead* – inner membrane complex, *e* – erythrocyte, *er* – rough endoplasmic reticulum, *l* – lipid droplet, *m* – mitochondrion, *n* – nucleus, *pr* – polar ring(s)*, small white arrow* – subpellicular microtubule, *v* – vacuole, *white arrow* – extensions of the parasitophorous vacuole membrane into the erythrocyte cytoplasm, *white arrowhead* – parasitophorous vacuole, *white asterisk* – apical pole of a budding merozoite, *white circle* – micropore.

**Secondary meronts** (Figs. 4A–4F) with budding merozoites were slightly smaller and exhibited a more electron-dense cytoplasm with few amylopectin granules compared to the stages of primary merogony. The merozoites were covered by a well-developed, three-layered pellicle (Fig. 4F) and displayed a prominent micropore in the anterior region (Fig. 4A). Their apical end contained the conoid, two preconoidal rings, two polar rings and subpellicular microtubules, micronemes, and a prominent rhoptry with a crystalline appearance (Figs. 4B, 4D, 4E). A section through one merozoite indicated that the subpellicular microtubules are arranged in a helical pattern (Fig. 4B). The nucleus of secondary merozoites was slightly irregular, somewhat elongated or bean-shaped, and surrounded by endoplasmic reticulum. The cytoplasm posterior to the nucleus contained several dense bodies and medium-sized, electron-lucent vacuoles (Fig. 4C). A large mitochondrion was seen in both the anterior and posterior regions of the developing merozoites. Solitary merozoites were observed after invading the host erythrocyte (Figs. 4G–4H). Their cytoplasm exhibited a similar electron density to that of budding secondary merozoites, with peripherally arranged micronemes in the anterior region, large lipid droplets, and a prominent mitochondrion. These forms appeared to lack amylopectin granules. Regularly spaced subpellicular microtubules were clearly visible in their tangential sections (Fig. 4G), and in some cases, a well-developed micropore could also be observed (Fig. 4H). The membrane of the parasitophorous vacuole surrounding solitary merozoites was strongly folded, with extensions into the cytoplasm of the host erythrocyte.

**Figure 5:**
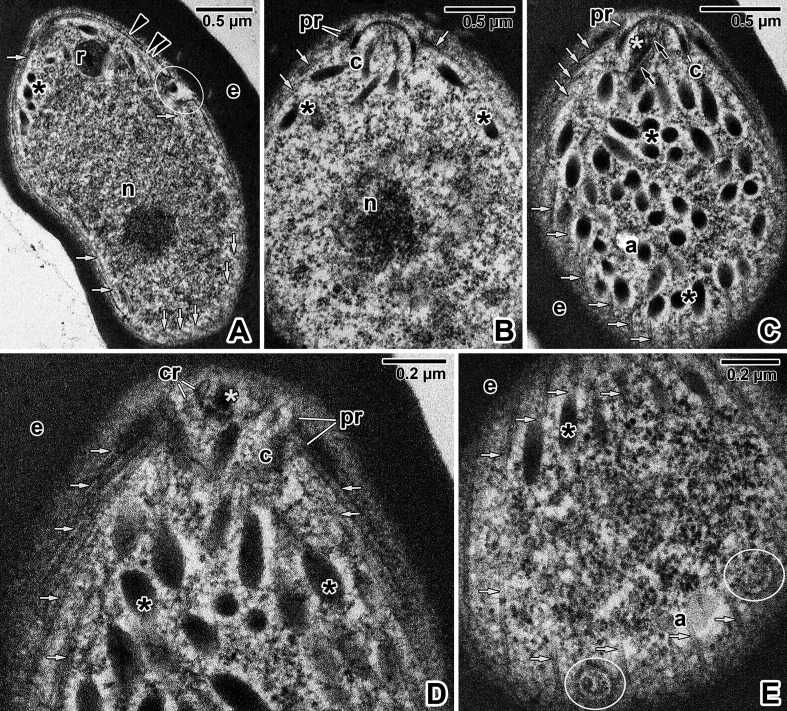
Subcellular organisation of *Dactylosoma* cf. *ranarum* SK secondary merozoites. A) Tangential section through a merozoite following erythrocyte invasion. Note the micropore (encircled) in its anterior region. B–C) Apical end of a secondary merozoite in mid-longitudinal (B) and superficial (C) sections. D–E) Tangentially sectioned anterior end of secondary merozoite showing the apical complex (D) and subpellicular microtubules with circular structures (encircled in E). Transmission electron microscopy. *a* – amylopectin granule, *black arrowhead* – parasite plasma membrane, *black asterisk* – microneme(s), *c* – conoid, c*r* – preconoidal rings, *double black arrowhead* – inner membrane complex, *e* – erythrocyte, *n* – nucleus, *pr* – polar ring(s), *r* – rhoptry, *small black arrow* – intraconoidal microtubule, *small white arrow* – subpellicular microtubule, *white asterisk* – rhoptry duct.

Occasionally, solitary intraerythrocytic stages resembling newly emerging trophozoites were observed; however, unlike trophozoites, they retained all organelles of the apical complex, including rhoptries and numerous micronemes surrounding the large nucleus (Figs. 5A–5E). The rhoptries exhibited a vesicular rather than crystalline appearance, and their size and density were reduced. The cytoplasm of these stages was granular but exhibited significantly lower electron density than that of budding merozoites or merozoites immediately after erythrocyte invasion and contained only a few amylopectin granules. Their anterior region was packed with numerous micronemes surrounding a large bean-shaped nucleus (Figs. 5A–5C). Subpellicular microtubules, emerging from the polar ring and extending posteriorly along the longitudinal cell axis, were clearly visible and appeared more numerous than in developing merozoites (Figs. 5C–5E). Based on the ultrastructural characteristics described above, we assume that these stages represent secondary merozoites undergoing transformation into gamonts. In surface sections of these parasites, unknown circular structures were observed at the level of subpellicular microtubules (Fig. 5E). It is not possible to determine whether these represent the lower part of cross-sectioned micropores containing host membrane coils or another unknown structure.

**Figure 6:**
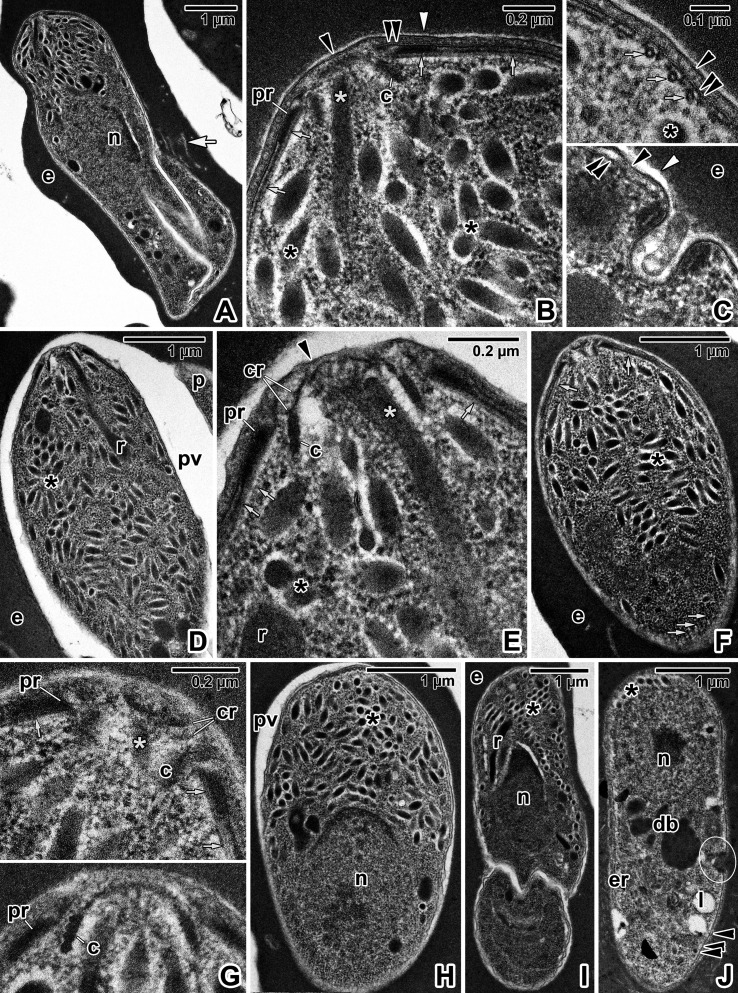
Subcellular organisation of *Dactylosoma* cf. *ranarum* SK gamonts. A) Anterior region of an immature gamont. B) Apical end of the gamont shown in A. C) Pellicle details showing subpellicular microtubules (top micrograph) and an active micropore with membrane structures filling its invaginated part (bottom micrograph). D) Anterior region of a more advanced stage of an immature gamont. E) Detail of the apical end of the gamont shown in D. F) Anterior region of a maturing gamont. G) Detailed views of the apical ends of maturing gamonts (top micrograph corresponds to the gamont shown in F). H) Anterior region of a mature gamont with a prominent nucleus and numerous micronemes. I) Longitudinal section of a recurved mature gamont in its folding region. J) Mid-posterior part of a mature gamont with a micropore (encircled) and several dense bodies. Transmission electron microscopy. *Black arrowhead* – parasite plasma membrane, *black asterisk* – microneme(s), *c* – conoid, cr – preconoidal rings, *db* – dense bodies, *double black arrowhead* – inner membrane complex, *e* – erythrocyte, *er* – rough endoplasmic reticulum, *n* – nucleus, *pr* – polar ring(s), *pv* – inner space of parasitophorous vacuole, *r* – rhoptry, *small white arrow* – subpellicular microtubule, *white arrow* – extensions of the parasitophorous vacuole membrane into the erythrocyte cytoplasm, *white arrowhead* – parasitophorous vacuole, *white asterisk* – rhoptry duct.

**Gamonts** (Figs. 6A–6J) were characterised by an electron-dense, grainy appearance, mainly due to abundant ribosomes filling their cytoplasm. The slightly flexed, immature gamonts were rather cylindrical to ovoid in shape and contained fewer organelles and a few amylopectin granules (Figs. 6A–6E). Micropores with a typical apicomplexan organisation, interrupting the inner membrane complex, were occasionally observed. An apparently active micropore was observed in one of the immature gamonts (Fig. 6C). As gamonts matured, they became more elongated and recurved within the erythrocyte (Fig. 6I). The number of organelles in their cytoplasm increased (Figs. 6D–6I), and dense bodies of various sizes appeared in the posterior region of mature gamonts (Fig. 6J). Amylopectin granules were observed only rarely. A large mitochondrion was also present, although it was not captured in the images shown. The well-developed apical complex contained the conoid with two preconoidal rings, prominent polar ring(s), few large rhoptries, and abundant micronemes (Figs. 6A–6B, 6D–6H). Regularly spaced, longitudinally oriented subpellicular microtubules associated with the polar ring were evident in the anterior region of all gamonts (Figs. 6B–6C, 6E).

### Molecular detection and phylogenetic analysis

All 56 newly obtained sequences from positive specimens were identical to each other and were assigned by BLAST to the unidentified *Dactylosoma* sp. [MN879399], previously isolated by Netherlands *et al*. [64] from a single specimen of *P. lessonae* collected in Belgium. Therefore, a single representative sequence (isolated from *P. ridibundus* collected in Devín) was chosen for further analyses. The newly obtained sequences were 99.84% identical (629/630 bp) to the *D. ranarum* sequences in GenBank [HQ224957–HQ224958]. The genetic similarity to other *Dactylosoma* species from GenBank was 99.36% to *D. kermiti* [MN879398], 98.92% to *D. piperis* [MW264134], and 97.37% to *D. amphibia* [OP480169].

**Figure 7:**
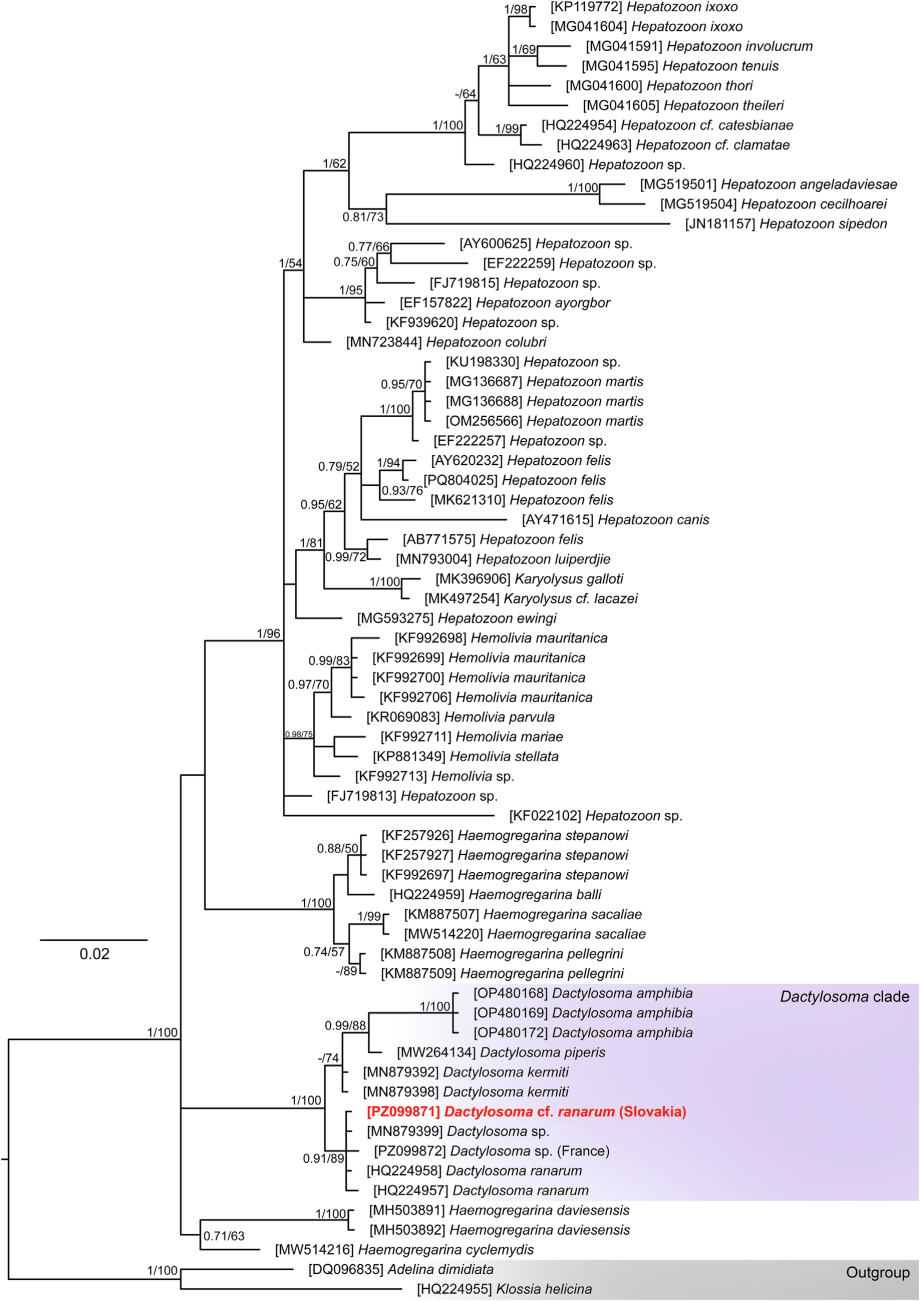
Phylogenetic tree of 66 sequences of various haemogregarine taxa reconstructed by Bayesian inference. The tree is based on a 614 bp-long alignment of ortholog partial 18S rDNA sequences and is rooted using *Adelina dimidiata* and *Klossia helicina* as outgroup. Values at the nodes indicate posterior probabilities (>70) from the Bayesian inference, and bootstrap values (>50) from the maximum likelihood analysis. Lower values are shown as dashes (–). The length of branches represents the number of substitutions per site. Newly generated sequence of *Dactylosoma* cf. *ranarum* SK collected from Slovakia is in red. GenBank accession numbers are in parentheses at the taxa name.

The final alignment used for the phylogenetic analyses included 66 ortholog sequences and spanned 614 unambiguous nucleotide positions. Both BI and ML reported similar tree topologies. Therefore, a consensus tree (Fig. 7) containing nodal support values for both analyses – posterior probability (PP) for BI and bootstrap support (BS) for ML – was generated. All analysed *Dactylosoma* sequences formed a well-supported monophyletic group with the unresolved position to the other analysed taxa. The *Dactylosoma* isolate from this study formed a moderately supported lineage (PP = 0.91; BS = 80) with *Dactylosoma* sp. from *P. lessonae* collected in Belgium, a newly sequenced *Dactylosoma* sp. from *P. esculentus* collected in France, and two sequences of *D. ranarum* from *P. esculentus* collected on Corsica. The sister lineage consisted of two species found in Brazilian anurans – *D. amphibia* from *Leptodactylus latrans, Rhinella diptycha*, and *Trachycephalus typhonius* (all sequences forming a monophyletic group) and *D. piperis* from *Leptodactylus labyrinthicus*. The position of sequences assigned to *D. kermiti* from *Ptychadena anchietae* and *Sclerophrys gutturalis* collected in South Africa within the *Dactylosoma* clade was not fully resolved.

### Evaluation of potential *Dactylosoma* dipteran vectors

In total, 454 mosquitoes representing seven species (*A. cantans*, *A. cinereus*, *A. sticticus*, *A. vexans*, *A. maculipennis*, *Cx. pipiens*, and *Cx. territans*) and 746 blackflies belonging to a single species (*S. balcanicum*) were collected and molecularly analysed for the presence of *Dactylosoma* (Table 2). Additional molecular screening was conducted on DNA extracted from pooled samples of 1,475 mosquitoes collected from various localities in western Slovakia, including frog sampling sites in Bratislava–Devín and Moravský Svätý Ján. The pooled mosquito samples represented five genera and eleven species (see Table 3 for the list of species and the number of individuals collected). No *Dactylosoma* spp. were detected in any of the examined dipteran species.

## Discussion

Currently, there are eight recognised species of *Dactylosoma*, with two of them described from fish hosts [21, 84]. The remaining six species were described from anuran hosts: (i) *D. kermiti* from the plain grass frog (*Ptychadena anchietae*) and the guttural toad (*Sclerophrys gutturalis*) in South Africa [64], (ii) *D. piperis* from the labyrinth frog (*Leptodactylus labyrinthicus*) in Brazil [100], (iii) *D. amphibia* from the cururu toad (*Rhinella diptycha*), veined tree frog (*Trachycephalus typhonius*), and Criolla frog (*Leptodactylus latrans*) in Brazil [101], (iv) *D. sylvatica* from the wood frog (*Boreorana sylvatica*) in Canada [21], (v) *D. taiwanensis* from the Alpine cricket frog (*Fejervarya limnocharis*) in Taiwan [56], and the type species (vi) *D. ranarum* first reported from *P. esculentus* in Italy [43], but subsequently documented in numerous other host species across Europe, Central and South America, Africa, and Asia [6, 12, 25, 54, 56, 72, 85, 88, 104, 106]. In addition, there are reports of unidentified species that could be assigned to *Dactylosoma*, albeit with varying degrees of certainty. These include, for example: (i) a haemogregarine morphologically conforming to *Dactylosoma* from toads collected in Brazil [19], (ii) *Dactylosoma* sp. isolated from *P. lessonae* collected in Belgium [64], and (iii) *Dactylosoma* sp. from Chaco granulated toad (*Rhinella major*) and can toad (*R. marina*) collected in Brazil [14]. Of these, only four recognised species (*D. amphibia*, *D. kermiti*, *D. piperis*, and *D. ranarum*) and a single unidentified *Dactylosoma* species from Belgium have been characterised molecularly, suggesting the potential existence of multiple distinct species.

Species previously classified under *Dactylosoma* include *Babesiosoma mariae* [33] and *B. jahni* [66]. Among species of uncertain affinity formerly considered *Dactylosoma* are *D. amaniae* found in the Fischer’s chameleon (*Kinyongia fischeri*) collected in West Africa [3] and *D. tritonis* described from the northern crested newt (*Triturus cristatus*) collected in an unspecified European country, likely the United Kingdom [20]. Neither species has ever been reported a second time, and both are more likely inclusions of rickettsial organisms based on their morphological descriptions [4]. Lastly, *D. clariae* infecting the African sharptooth catfish (*Clarias gariepinus*) [31] and *D. tilapiae* from unspecified Nile tilapia [34] both collected in Egypt lack sufficient evidence to confirm their validity as distinct species [62].

Prior to this study, there were no published records of *Dactylosoma* or other blood apicomplexans in Slovak anurans. In a survey of 239 individuals representing nine anuran species from western Slovakia, *Dactylosoma* was detected in three *Pelophylax* species (*P. esculentus*, *P. ridibundus*, and *P. lessonae*), with an overall prevalence of 40.85% (67/164). Given the absence of examined type material and the lack of comparative molecular data from specimens collected at or near the type locality, we conservatively assign the material examined here to *Dactylosoma* cf. *ranarum*.

### Molecular identification

In the analysed short 18S rDNA region, the *Dactylosoma* in this study were genetically identical to the previously recorded *Dactylosoma* sp. from Belgium [64]. Although the authors of the former study hypothesised its identity to be *D. ranarum*, an interspecific divergence of 0.2% was recorded between Belgian *Dactylosoma* sp. and the sequences of *D. ranarum* obtained from Corsica. Several other haemogregarines also exhibit similarly low interspecific divergence, namely *Hepatozoon clamatae* and *H. catesbianae*, and *H. chinensis* and *H. ayorgbor* (both pairs with a divergence of 0.3%). The 18S rDNA sequence of *D.* cf. *ranarum* SK differs marginally from the sequences designated in GenBank as *D. ranarum* [HQ224957–HQ224958], which may represent intraspecific genetic variability. Unfortunately, these are the only *D. ranarum* sequences available to date [9], published without any morphological identification of the parasite (*i.e.* nonugens – GenBank sequences unaccompanied by morphological evidence).

While the 18S rDNA gene serves as a common molecular marker for a broad range of apicomplexan parasites, including haemogregarines, it has been shown to possess limited resolution between closely related species within the suborder Adeleorina [9]. The gene provides sufficient information on the relationships between different genera, however, it is less informative for species differentiation. Furthermore, several studies have documented very low interspecific divergence within various haemogregarine genera, with particularly low values observed among *Dactylosoma* spp. [64, 100]. This often complicates accurate identification and classification of haemogregarine species. In haemogregarines, three genomes are present, two of which are extrachromosomal: one mitochondrial and one apicoplast [22, 50]. Apicomplexan mitochondrial genomes are small compared to those of most other eukaryotes (6–7 kb) and have a conserved structure, containing three protein-coding genes: COI, cytochrome c oxidase subunit III (COIII), and cytochrome *b* (CytB), along with various RNA fragments. The complete mitochondrial genome of *H. catesbianae* was sequenced by Léveillé *et al.* [49], but similar work on *Dactylosoma* is still lacking. In anuran *Hepatozoon* spp., interspecific pairwise differences in COI, COIII, and CytB range from 0.3–1.8% [50], whereas *Haemogregarina* spp. from turtles in Colombia, showed up to 24.0% intraspecific variability in these genes, thus the authors hypothesised that above 30% genetic distance in mitochondrial markers might be associated with different parasite species [30]. The potential of individual mitochondrial protein-coding genes as genetic markers for different anuran blood apicomplexans, relative to the whole mitogenome, has yet to be assessed, and although these regions can be highly variable in some taxa, they may still preserve valuable phylogenetic signals [30]. However, no mitochondrial genome data are currently available for *Dactylosoma*, and future research should prioritise obtaining such data to help resolve taxonomic uncertainties in this group.

### Morphology, life cycle, and vectors

In blood smears from *Pelophylax* frogs, we observed all developmental stages of *Dactylosoma* known to occur in vertebrate peripheral blood, including trophozoites, primary and secondary merogony stages, and gamonts. The phenotypic characteristics of *D.* cf. *ranarum* SK are typical of the genus and are consistent with previously published light microscopic observations on *Dactylosoma* spp. (*e.g*., [4, 64, 100, 101]). Interestingly, all developmental stages of *D.* cf. *ranarum* SK exhibited variability in the intensity and shade of Giemsa staining. However, it should not be considered a reliable characteristic, as differences in Giemsa staining are commonly observed between smears prepared in different laboratories. As illustrated in our photodocumentation, smears prepared in Bratislava stained an intense pink (*e.g*. top micrographs in Figs. 1K and 1P), whereas smears prepared in Brno showed a more blue-violet hue (e.g. bottom micrographs in Figs. 1K and 1P). These discrepancies are typically attributable to the use of Giemsa stains from different batches or manufacturers, or to differences in the properties of tap water used for rinsing (*e.g.* Brno tap water has higher hardness). *Dactylosoma* also appears to exhibit considerable variability in the number of merozoites produced during both primary and secondary merogony, even within the same species, as observed in this study. This variability represents one of the main sources of uncertainty in *D. ranarum* identification (see Remarks and differential diagnosis). Such variations during merogony, resulting in different numbers of merozoites produced, have also been observed in other apicomplexans [58].

The differentiation between primary and secondary merogony stages in this study was based on a combination of morphological and quantitative characters observed consistently across multiple specimens and supported by comparison with published data. Primary meronts were generally larger, more variable in shape, and contained more nuclei (up to 16, most often 12), whereas secondary meronts were smaller, with fewer nuclei (up to 8) typically arranged on one side of the cell. Primary meronts also showed greater variability in cytoplasmic structure, with young stages often containing numerous vacuoles and non-staining inclusions of various sizes, whereas secondary meronts were more uniform and frequently exhibited a fan-like or dactylate morphology. Differences between merozoites were subtler; however, secondary merozoites were less numerous (up to 8 vs. up to 16) and tended to contain more prominent cytoplasmic vacuoles.

The subcellular organisation of *D.* cf. *ranarum* SK generally corresponds to that described in previous studies [4, 5, 12]. It should be noted that determining the type of merogony in ultrathin sections requires sufficient material for comparison and may not be completely unambiguous. Except for the absence of amylopectin granules in primary merozoites and differences in cytoplasmic density reported by Barta [4], the criteria used in previous studies [4, 5, 12] to determine the type of merogony remain unclear. Although Boulard *et al.* [12] claimed to describe type primary meronts with developing merozoites, and Barta *et al.* [4, 5] focused on secondary merogony, published electron micrographs do not reveal any clear differences that would allow for an unambiguous distinction between primary and secondary merogony, with the merogonic stages depicted in their studies appearing nearly identical.

In this study, we evaluated the TEM data in relation to previously published ultrastructural studies and our own light microscopy observations. We classified the less electron-dense meronts with budding merozoites that lack amylopectin granules as stages of primary merogony, while the slightly smaller, more electron-dense stages with merozoites containing a few amylopectin granules, along with electron-dense bodies and several vacuoles in their cytoplasm, were classified as stages of secondary merogony. The subcellular organisation of merogonic stages of both generations in this study corresponded to that reported in previous studies [4, 5, 12], including the presence of a prominent rhoptry with a crystalline appearance. Furthermore, we observed two types of solitary intracellular zoite stages: one with an electron-dense appearance, corresponding to a secondary merozoite shortly after erythrocyte invasion, and another with a markedly more electron-translucent appearance. We assume these represent secondary merozoites in the process of transforming into gamonts. Although we cannot explain the occurrence of a temporary reduction in cytoplasmic density during this transformation, light-microscopic observations of overgrown merozoites and immature gamonts with whitish (hyaline) cytoplasm in Giemsa-stained smears support this hypothesis. We ruled out the possibility that these were primary merozoites transforming into trophozoites (which share a similar cytoplasmic density), as they retained a complete apical complex and contained more micronemes than either type of merozoite. The presence of rhoptries reduced in size and electron density indicates that they were discharging during the invasion process [5]. The fine structure of *D.* cf. *ranarum* SK gamonts, with immature stages possessing fewer developed organelles than mature gamonts, which are equipped with numerous micronemes, rhoptries, and dense granules, is consistent with previous studies on *D. ranarum* [4, 5]. The organisation of trimembrane pellicle and the arrangement of subpellicular microtubules and conoid-associated microtubules observed at several developmental stages are also consistent in all these studies. In both budding and solitary merozoites (Figs. 3G, 4D, 5B, 5D), we observed a pair of polar rings, as also reported for *D. ranarum* secondary merozoites and young gamonts in previous works [4, 5]. While these stages of *Dactylosoma* possess two polar rings, similar to other apicomplexans such as *Lankesterella* sporozoites [87, 92] and *Toxoplasma* tachyzoites [41], maturing and mature gamonts ([5], this study]) instead exhibit a broad, dense structure in the polar ring region (Figs. 6B, 6E, 6G), resembling (though less massive than) the “polar ring complex” described in *Haemogregarina* gamonts [70]. It has been suggested that the polar ring complex and conoid, present in motile stages undergoing sporogony in invertebrate hosts, represent primitive features adapted to facilitate parasite movement through host tissues [70].

Although these ultrastructural features are not yet sufficient for species-level diagnosis on their own, they provide a valuable comparative framework for *Dactylosoma* taxonomy. In practice, the most informative characters appear to include the organisation of the apical complex, with particular emphasis on the polar ring(s); the position and activity of micropores; the number of subpellicular microtubules; and rhoptry morphology, particularly the presence of crystalline contents. Additional useful characters include the occurrence and abundance of amylopectin granules and dense bodies in merozoites and gamonts. Furthermore, the degree of elaboration of the parasitophorous vacuole membrane within the host erythrocyte may offer useful taxonomic clues [4, 5, 12]. At present, however, these features should be regarded as supportive rather than definitive. Ultrastructural data are available for too few *Dactylosoma* species to determine which traits are stable at the species level and which are conserved more broadly across the genus. Importantly, most previous ultrastructural studies of *Dactylosoma* spp. were conducted without accompanying molecular data, limiting their applicability for species-level taxonomy. In contrast, the present study integrates ultrastructural observations with molecular characterisation, enabling internal morphological features to be linked to a defined genotype. This integrative approach provides a more robust framework for future taxonomic comparisons, as ultrastructural characters can now be interpreted in the context of genetically confirmed lineages rather than relying solely on morphology.

In contrast to the intraerythrocytic development of *Dactylosoma* parasites in vertebrate hosts, which is relatively well characterised, their development in natural vectors has yet to be elucidated. However, there have been several experimental attempts to identify their possible vectors. Some scientists speculated that leeches may serve as vectors, originally acting as the primary hosts of *Dactylosoma*, in which the parasite underwent a monoxenous and simpler life cycle [56]. Initial experiments attempting to transmit *D. ranarum* to other invertebrates, such as Eurasian glossiphoniid leech (*Hemiclepsis marginata*) and European biting midge (*Culicoides nubeculosus*), proved unsuccessful [12, 67]. In another experiment, *P. esculentus* from Corsica with natural infection with *D. ranarum* was used to transmit the parasite to a North American glossiphoniid leech (*Desserobdella picta*), a natural vector of the closely related *Babesiosoma stableri* [4, 8]. The parasite apparently underwent sporogony in the intestinal epithelium of the leech and the observed oocysts were polysporoblastic, producing 30 or more sporozoites through exogenous budding directly into the cytoplasm of the leech enterocyte. The sporozoite production was not synchronised, as parasitised cells demonstrated fully mature sporozoites and sporonts simultaneously [4, 5]. Nevertheless, no gamete development or zygote formation were observed, and attempts to experimentally transmit the parasite to an uninfected frog host were unsuccessful [4]. Since both studies suggested the potential of leeches to transmit dactylosomatid parasites, they were widely believed to serve as the sole vectors of *Dactylosoma* parasites. Interestingly, Nöller [67] suggested that the fish louse (*Argulus foliaceus*) could be a possible vector of *Dactylosoma* in frogs, as it was the only blood-sucking ectoparasite – apart from the leech *Hemiclepsis* – observed to infest frogs and tadpoles in the pond at the collection site.

Despite the numerous similarities, however, there are also indications that the life cycle and transmission strategies may differ between *Babesiosoma* and *Dactylosoma*. For example, compared to *B. stableri* [7], only a small amount of amylopectin inclusions was observed in both *D.* cf. *ranarum* SK and *D. ranarum* [5]. The number of amylopectin granules in *B. stableri* increased over the course of the infection, with mature gamonts containing large stores of amylopectin [7]. The authors suggested that the large reserves of energy in the form of amylopectin and lipid inclusions could be consumed during sporogonic development in the leech. The difference in amounts of amylopectin granules between the two genera could reflect their different lifespans within the anuran host or transmission strategy. While the gamonts of *B. stableri* can survive in the blood for approximately a year without reinfection [86], the gamonts of *D. ranarum* have been observed in frog blood for a maximum of several weeks [5].

A more recent study revealed the development of *D. kermiti* in two species of African mosquitoes, *Uranotaenia mashonaensis* and *U. montana*, which were observed feeding on parasitised frogs *in situ* [64]. Subsequent examination of smears from their guts and haemocoels unveiled putative developmental stages of *D. kermiti*, including gametes, ookinetes, oocysts, and sporozoites. This finding, therefore, suggests the involvement of dipterans in *Dactylosoma* transmission. The absence of the parasite in the mosquitoes’ salivary glands implies that *D. kermiti* may be transmitted to frog hosts through ingestion of the infected dipteran rather than through its bite, as observed in other haemogregarines. However, the authors themselves point out that the study is preliminary and based on limited data, which highlights the need for further observations to draw definitive conclusions about the parasite life history [64]. To elucidate the complete life cycle of *D.* cf. *ranarum* SK, including its potential insect vectors, we carefully examined blood smears from all sampled anurans and performed molecular detection of *Dactylosoma* in all blood-sucking dipterans collected from the same localities. Although we were able to repeatedly detect and document all developmental stages of *Dactylosoma* in the blood of its vertebrate host, molecular analysis did not reveal the presence of the parasite in any of the collected mosquitoes. This result is unsurprising, as *Aedes* mosquitos – comprising most of our samples from Devín and Rusovce – are opportunistic feeders that primarily target mammalian hosts, including humans. This feeding behaviour makes them important vectors of human viral diseases such as dengue fever, yellow fever, and Zika virus [52], but it also renders them highly unlikely to feed on poikilothermic animals. It is therefore plausible that frog haemoparasites are transmitted by other mosquito species, such as *Cx. territans*, which is rare in our region, or by mosquitoes of the genus *Uranotaenia*, which is so far absent in Slovakia [69]. *Culex territans*, commonly known as the northern frog-biting mosquito, is found across North America, Europe, North Africa, and the Arabian Peninsula, typically inhabiting clean, vegetated aquatic environments. While females can feed on various vertebrates, they predominantly target reptiles and amphibians, with a particular preference for frogs [11]. Moreover, *Cx. territans* has previously been implicated as a vector of anuran trypanosomes [10], haemogregarines of the genus *Hepatozoon* [38], and the fungus *Batrachochytrium dendrobatidis* [79], making it a potential candidate for *Dactylosoma* transmission. However, although the presence of *Cx. territans* has been documented in Slovakia [46, 93], we did not collect this species at Devin and Rusovce, and its occurrence at these sampling sites remains unknown. To test the possibility that this mosquito could serve as a vector for the parasite studied here, we included in the molecular screening for *Dactylosoma* frog blood and mosquito samples collected in Moravský Svätý Ján, where *Cx. territans* had been recorded in previous years (personal unpublished data). Nevertheless, we captured only four female specimens of *Cx. territans*, all of which tested negative for *Dactylosoma*. In contrast, female blackflies are known to bite exclusively warm-blooded hosts, as their mouthparts and host-seeking behaviour are specifically adapted to endothermic animals [94]. They were included in the molecular screening solely because of their high numbers in traps set at Devín and Rusovce, particularly in light of the negative results obtained from mosquitoes. As expected, *Dactylosoma* was not detected in any of the blackfly samples.

In conclusion, while leeches have not yet been confirmed as natural vectors for any *Dactylosoma* species, their involvement in the transmission of amphibian haemoparasites cannot be excluded. This is especially true for semi-aquatic hosts such as *Pelophylax*, which is the only anuran genus in which *Dactylosoma* has been detected during our studies in Slovakia to date. In Europe, several leech species feed on amphibians, including *Batracobdella algira* [53], *Helobdella stagnalis* [98], *marginata* [55], and *Hirudo medicinalis* [59]. According to Vladimír Košel from Comenius University in Bratislava (personal communication, 2022), of these species, only *H. stagnalis* is known to occur at the studied localities, making it a potential vector of *Dactylosoma* in western Slovakia and a suitable focus for future studies.

### Host-parasite interactions and infection parameters

Although *Dactylosoma* is generally assumed to have minimal effects on host blood cells, blood smears and ultrathin sections from infected frogs often revealed numerous degenerated or destroyed erythrocytes. Characteristic intraerythrocytic stages of *Dactylosoma*, including trophozoites, meronts, and immature gamonts, were often observed in close association with damaged erythrocytes or embedded within them. Immature and mitotic erythrocytes are commonly observed in the peripheral blood of amphibians and may represent a physiological response to blood parasite infection [23, 26]. Infection with haemogregarines (including *Dactylosoma*) may induce anaemia in birds, reptiles, and amphibians [26, 109]. For example, haemogregarine-infected *Litoria infrafrenata* frogs lacking apparent signs of disease showed changes in haematologic and biochemical variables, suggesting subclinical impact of infection on health status [109].

*Dactylosoma* cf. *ranarum* SK exhibited a relatively high overall prevalence in *Pelophylax* water frogs, with the highest occurrence in *P. ridibundus* and *P. esculentus* and a notably lower presence in *P. lessonae*. This pattern is consistent with the generally higher prevalences reported for this species compared to other *Dactylosoma* spp., although it remains below the highest recorded overall prevalence observed for *D. ranarum* in *P. esculentus* from Corsica, which reached 53.49% (46/86) [6]. Netherlands *et al.* [64] reported an overall prevalence of 30.04% (70/233) for *D. kermiti* in two host species collected in KwaZulu-Natal, South Africa. Regarding *Dactylosoma* species found in Brazilian anurans: (i) *D. piperis* was found in the only collected and screened individual of *L. labyrinthicus* [100], (ii) *D. amphibia* had an overall prevalence of 2.59% (3/116) across three host species [101], and (iii) the unidentified species of *Dactylosoma* reported by Coêlho *et al.* [14] had a prevalence of 6.85% (5/73) across two host species.

Similarly, the level of parasitaemia of *D.* cf. *ranarum* SK with an average parasitaemia of 2.3% in *P. ridibundus* (*n* = 11) and 1.7% in *P. esculentus* (*n* = 45), was significantly higher than in other studies. For example, parasitaemia of 0.3% was reported for the *Dactylosoma* sp. from a single individual of *P. lessonae*, 0.4% for *D. kermiti* (*n* = 70), 0.2% for *D. piperis* (*n* = 1), and 0.1% for *D. amphibia* (*n* = 3) [64, 100, 101].

The higher levels of *D. ranarum* prevalence and parasitaemia compared to other studies on *Dactylosoma* may result from the different ecology and behaviour of the hosts. The frogs of the genus *Pelophylax* are considered semi-aquatic, inhabiting diverse freshwater environments. This, coupled with potentially high population densities, provides ample opportunity for infections and pathogen transmission between hosts. For example, *Pelophylax* water frogs in Europe are indeed recognised for harbouring a wide range of pathogens compared to some other anuran species [40, 44, 83]. Moreover, semi-aquatic and aquatic anurans in South Africa exhibit higher prevalence of haemoparasites compared to terrestrial species [65]. This hypothesis is further supported by reports of the highest prevalence of *Dactylosoma* to date, which detected the parasites specifically in *Pelophylax* representatives ([6], this study). It is therefore likely that the different ecology and behaviour of various investigated anuran species could be the reason why we did not find *D.* cf. *ranarum* SK in other anurans investigated during this study. More frequent encounters of *Pelophylax* spp. with vectors may also lead to reinfections, resulting in increased parasitaemia. This is especially true if *Dactylosoma* is transmitted by leeches. Outside the breeding season, members of other genera screened here typically inhabit more terrestrial habitats compared to *Pelophylax* frogs, hereby reducing their contact with potential vectors, such as leeches and mosquitoes commonly found in or near water sources. Additionally, some of these species (*e.g. Bufo* spp. and *Bombina* spp.) are known to produce toxins in their skin, which can be irritating or toxic to microbes and predators [18, 89], which reduces the likelihood of bites by potential vectors and thus their exposure to blood parasites.

Due to the drying of the water source in Devín, we could only assess the effect of sampling month on parasitaemia in *P. esculentus* (*n* = 33) collected in Rusovce. Seasonal analysis showed that all epidemiological parameters rose from moderate levels in April (spring) to peaks in June (summer), then declined to their lowest levels by August (summer). The spring–summer period coincides with peak vector activity, increasing host exposure to infection, while by late summer, vector populations may decline due to natural controls such as predation or desiccation of breeding sites [17, 61]. Haemogregarine infections of frogs and tortoises were shown to be higher in wet seasons, reflecting vector activity, whereas dry-season sampling showed no seasonal effect [1, 78, 109]. No studies have explored seasonal effects on anuran haemoparasites in Europe, and no clear overall trend was observed in the prevalence of *B. stableri*, a close relative of *Dactylosoma*, in bullfrogs monitored over a three-year period in Canada [4]. As ectotherms, amphibians are strongly influenced by temperature and humidity, which can affect both parasite development and host immune function, contributing to seasonal infection patterns [32]. Post-hibernation, their immunity is weakened, and summer stressors such as heat, breeding, and predation further tax defences [28, 57, 75], while stronger immunity in late summer may reduce parasite loads. Habitat shifts, breeding behaviour, and host density can also affect transmission. Although it is unclear whether *Dactylosoma* overwinters in vectors or hosts, high early-season values in our study suggest frogs may retain parasites from the previous season. Host species or sex effects could not be assessed here, as all infected frogs belonged to a single species (*P. esculentus*), 85% of which were females. However, another study on haemogregarine infection in *Pelophylax* frogs found no significant differences between the sexes, although it did confirm a significant difference between adults and subadults [71]. In contrast, haemogregarine infection in wall lizards was shown to be slightly lower in females in summer than in spring, while infection levels in males remained consistent. The authors attributed this pattern to the immunosuppressive effects of testosterone, combined with greater exposure to vectors due to increased male mobility [2]. Overall, multiple factors interact to shape seasonal parasite prevalence patterns, including host and parasite species, parasite life cycle, vector activity, host ecology and biology, host sex and age/size, seasonal changes in host behaviour and immunity, and environmental conditions [1, 16, 35, 71, 76, 78, 109].

## Conclusions

Observations of *Dactylosoma* in three species of *Pelophylax* water frogs from western Slovakia and comparison with the *Dactylosoma* isolate from *P. esculentus* collected in France (Perpignan) suggest that the species from the current study represent a single species corresponding morphologically to the type species *D. ranarum*, and identical to the *Dactylosoma* sp. sequence [GenBank accession number MN879399] previously recorded from *P. lessonae* in Belgium. However, given the considerable variability in the number of final merozoites produced during primary and secondary merogony, as well as in the dimensions of all developmental stages, and the absence of molecular data in the original description and older studies, it cannot be excluded that what is currently regarded as a single species may in fact represent a species complex. Future studies should incorporate data from across Europe, including material from the type locality, to help resolve the question posed above.

## Data Availability

The data underlying this article are available in the article and newly generated *Dactylosoma* cf. *ranarum* SK sequences are available in GenBank (under accession numbers PZ099871 and PZ099872).

## References

[1] Adetunji VE, Ogundipe GT, Adeyemo OK. 2022. Prevalence and parasite intensity of haemogregarines in African bell hinge-back (*Kinixys belliana*) and African home's hinge-back (*Kinixys homeana*) tortoises in Ibadian, Nigeria. Journal of Wildlife Diseases, 58, 825–835. 36321922 10.7589/JWD-D-21-00084

[2] Amo L, López P, Martín J. 2005. Prevalence and intensity of haemogregarine blood parasites and their mite vectors in the common wall lizard, *Podarcis muralis*. Parasitology Research, 96, 378–381. 15940525 10.1007/s00436-005-1354-2

[3] Awerinzew SV. 1914. Beiträge zur Morphologie und Entwicklungsgeschichte der Protozoen von Deutsch-Ost-Afrika. Journal de Microbiologie, 1, 1–10. (In Russian, German Summary).

[4] Barta JR. 1991. The Dactylosomatidae, in Advances in Parasitology, Baker JR, Muller R, Editors. Academic Press. p. 1–37. 10.1016/s0065-308x(08)60305-x 2069071

[5] Barta JR, Boulard Y, Desser SS. 1987. Ultrastructural observations on secondary merogony and gametogony of *Dactylosoma ranarum* Labbé, 1894 (Eucoccidiida; Apicomplexa). Journal of Parasitology, 73, 1019–1029.

[6] Barta JR, Boulard Y, Desser SS. 1989. Blood parasites of *Rana esculenta* from Corsica: comparison of its parasites with those of Eastern North American ranids in the context of host phylogeny. Transactions of the American Microscopical Society, 108, 6–20.

[7] Barta JR, Desser SS. 1986. Light and electron microscopic observations on the intraerythrocytic development of *Babesiosoma stableri* (Apicomplexa, Dactylosomatidae) in frogs from Algonquin Park, Ontario. Journal of Protozoology, 33, 359–368.

[8] Barta JR, Desser SS. 1989. Development of *Babesiosoma stableri* (Dactylosomatidae; Adeleida; Apicomplexa) in its leech vector (*Batracobdella picta*) and the relationship of the dactylosomatids to the piroplasms of higher vertebrates. Journal of Protozoology, 36, 241–253.

[9] Barta JR, Ogedengbe JD, Martin DS, Smith TG. 2012. Phylogenetic position of the adeleorinid coccidia (Myzozoa, Apicomplexa, Coccidia, Eucoccidiorida, Adeleorina) inferred using 18S rDNA sequences. Journal of Eukaryotic Microbiology, 59, 171–180. 22313415 10.1111/j.1550-7408.2011.00607.x

[10] Bartlett-Healy K, Crans W, Gaugler R. 2009. Vertebrate hosts and phylogenetic relationships of amphibian trypanosomes from a potential invertebrate vector, *Culex territans* Walker (Diptera: Culicidae). Journal of Parasitology, 95, 381–387. 18850768 10.1645/GE-1793.1

[11] Bhosale CR, Burkett-Cadena ND, Mathias D. 2023. Northern frog biting mosquito *Culex territans* (Walker 1856) (Insecta: Diptera: Culicidae): EENY-803 IN1394, 3. EDIS 2023, 2023(2). Gainesville, FL.

[12] Boulard Y, Vivier E, Landau I. 1982. Ultrastructure de *Dactylosoma ranarum* (Kruse, 1890); affinités avec les coccidies; révision du statut taxonomique des dactylosomides. Protistologica, 18, 103–121.

[13] Celli A, San Felice F. 1891. Über die Parasiten des roten Blutkörperchens im Menschen und in Tieren. Fortschritte der Medizin, 9, 499–511.

[14] Coêlho TA, De Souza DC, da Costa Oliveira E, Correa LL, Viana LA, Kawashita-Ribeiro RA. 2021. Haemogregarine of genus *Dactylosoma* (Adeleorina: Dactylosomatidae) in species of *Rhinella* (Anura: Bufonidae) from the Brazilian Amazon. Acta Parasitologica, 66, 1574–1580. 33997935 10.1007/s11686-021-00399-z

[15] Cook CA, Netherlands EC, Smit NJ. 2015. First *Hemolivia* from southern Africa: reassigning chelonian *Haemogregarina parvula* Dias, 1953 (Adeleorina: Haemogregarinidae) to *Hemolivia* (Adeleorina: Karyolysidae). African Zoology, 50, 165–173.

[16] Dajčman U, Carretero MA, Megía-Palma R, Perera A, Kostanjšek R, Žagar A. 2022. Shared haemogregarine infections in competing lacertids. Parasitology, 149, 193–202. 35234602 10.1017/S0031182021001645 PMC11010482

[17] Dambach P. 2020. The use of aquatic predators for larval control of mosquito disease vectors: Opportunities and limitations. Biological Control, 150, 104357.

[18] Dulger B, Tok C, Kaya S, Sevinc M. 2006. Antimicrobial activity in the skin secretion of *Hyla arborea* (Linnaeus, 1758). Russian Journal of Herpetology, 14, 117–120.

[19] Durham HE. 1902. Report of the yellow fever expedition to Parà of the Liverpool School of Tropical Medicine and Medical Parasitology. Longmans, Green for the University Press of Liverpool: London.

[20] Fantham HB. 1905. *Lankesterella tritonis* n. sp., a haemogregarine from the blood of the newt, *Triton cristatus* (*Molge cristata*). Zoologischer Anzeiger, 29, 257–263.

[21] Fantham HB, Porter A, Richardson LR. 1942. Some Haematozoa observed in vertebrates in eastern Canada. Parasitology 34, 199–226.

[22] Feagin JE. 1994. The extrachromosomal DNAs of apicomplexan parasites. Annual Review of Microbiology, 48, 81–104. 10.1146/annurev.mi.48.100194.000501 7826027

[23] Forzán MJ, Heatley J, Russell KE, Horney B. 2017. Clinical pathology of amphibians: a review. Veterinary Clinical Pathology, 46, 11–33. 28195641 10.1111/vcp.12452

[24] Forzán MJ, Vanderstichel RV, Ogbuah CT, Barta JK, Smith TG. 2012. Blood collection from the facial (maxillary)/musculo-cutaneous vein in true frogs (family Ranidae). Journal of Wildlife Diseases, 48, 176–180. 22247387 10.7589/0090-3558-48.1.176

[25] Gaibova HD, Mamedova SO. 2010. Blood parasites of the Eurasian marsh frog Pelophylax ridibundus from the fresh waters of Azerbaijan. Journal of V.N. Karazin Kharkiv National University, 12, 54–60.

[26] González LP, Vargas-León CM, Fuentes-Rodríguez GA, Calderón-Espinosa ML, Matta NE. 2021. Do blood parasites increase immature erythrocytes and mitosis in amphibians? Revista de Biología Tropical, 69, 615–624.

[27] Grassi B, Feletti R. 1892. Contribuzione allo studio dei parassiti malarici. Accademia Gioenia di Scienze Naturali in Catania, 5, 1–85.

[28] Groner ML, Rollins-Smith LA, Reinert LK, Hempel J, Bier ME, Relyea RA. 2014. Interactive effects of competition and predator cues on immune responses of leopard frogs at metamorphosis. Journal of Experimental Biology, 217, 351–358. 24115058 10.1242/jeb.091611

[29] Günther R. 1990. Die Wasserfrösche Europas (Anura - Froschlurche). Ziemsen: Wittenberg Lutherstadt.

[30] Gutierrez-Liberato GA, Lotta-Arévalo IA, González LP, Vargas-Ramírez M, Rodríguez-Fandiño O, Cepeda AS, Ortiz-Moreno ML, Matta NE. 2021. The genetic and morphological diversity of *Haemogregarina* infecting turtles in Colombia: Are mitochondrial markers useful as barcodes for these parasites? Infection, Genetics and Evolution, 95, 105040. 10.1016/j.meegid.2021.105040 34403833

[31] Haiba MH. 1962. On the Nile fish parasites in Egypt, *Cytauxzoon clariae* n. sp. from the Egyptian Nile fish, *Clarias lazera*. Journal of the Arab Veterinary Medical Association, 22, 249–256.

[32] Herczeg D, Ujszegi J, Kásler A, Holly D, Hettyey A. 2021. Host–multiparasite interactions in amphibians: a review. Parasites & Vectors, 14, 296. 34082796 10.1186/s13071-021-04796-1 PMC8173923

[33] Hoare CA. 1930. On a new *Dactylosoma* occurring in fish of Victoria Nyanza. Annals of Tropical Medicine & Parasitology, 24, 241–248.

[34] Imam EA, Marzouk MSM, Hassan AA, Derhall YFS, Itman RH. 1985. Studies on blood parasites in Nile fishes. Journal of the Egyptian Veterinary Medical Association, 2, 97–108.

[35] Isaak Delgado AB, Zavala-Norzagaray AA, Espinoza-Romo BA, Ortega-Anaya JG, Ley-Quiñonez CP, Aguirre A, Rendón-Franco E. 2023. Hematologic parameters and the effect of hemoparasites of wild anurans in Northern Sinaloa, Mexico. Veterinary Clinical Pathology, 52, 386–395. 37127551 10.1111/vcp.13214

[36] Katoh K, Rozewicki J, Yamada KD. 2019. MAFFT online service: multiple sequence alignment, interactive sequence choice and visualization. Briefings in Bioinformatics, 20, 1160–1166. 28968734 10.1093/bib/bbx108 PMC6781576

[37] Kearse M, Moir R, Wilson A, Stones-Havas S, Cheung M, Sturrock S, Buxton S, Cooper A, Markowitz S, Duran C, Thierer T, Ashton B, Meintjes P, Drummond A. 2012. Geneious Basic: An integrated and extendable desktop software platform for the organization and analysis of sequence data. Bioinformatics, 28, 1647–1649. 22543367 10.1093/bioinformatics/bts199 PMC3371832

[38] Kim B, Smith TG, Desser SS. 1998. The life history and host specificity of *Hepatozoon clamatae* (Apicomplexa: Adeleorina) and ITS-1 nucleotide sequence variation of *Hepatozoon* species of frogs and mosquitoes from Ontario. Journal of Parasitology, 84, 789–797. 9714212

[39] Knoz J. 1965. To identification of Czechoslovakian black flies (Diptera, Simuliidae). Folia Facultatis Scientiarium Naturalium Universitatis Masarykianae Brunensis, Biologia, 6, 1–56.

[40] Kolenda K, Najbar A, Ogielska M, Baláž V. 2017. *Batrachochytrium dendrobatidis* is present in Poland and associated with reduced fitness in wild populations of *Pelophylax lessonae*. Diseases of Aquatic Organisms, 124, 241–245. 28492180 10.3354/dao03121

[41] Koreny L, Zeeshan M, Barylyuk K, Tromer EC, van Hooff JJE, Brady D, Ke H, Chelaghma S, Ferguson DJP, Eme L, Tewari R, Waller RF. 2021. Molecular characterization of the conoid complex in *Toxoplasma* reveals its conservation in all apicomplexans, including *Plasmodium* species. PLoS Biology, 19, e3001081. 33705380 10.1371/journal.pbio.3001081 PMC7951837

[42] Kramář J. 1958. Fauna ČSR, svazek 13: Komáři bodaví – Culicinae (Řád: Dvoukřídlí-Diptera). Nakladatelství Československé akademie věd: Praha.

[43] Kruse W. 1890. Ueber Blutparasiten. Archiv für pathologische Anatomie und Physiologie und für klinische Medicin, 120, 541–560. PMC5182399 30163712

[44] Kuzmin Y, Dmytrieva I, Marushchak O, Morozov-Leonov S, Oskyrko O, Nekrasova O. 2020. Helminth species and infracommunities in frogs *Pelophylax ridibundus* and *P. esculentus* (Amphibia: Ranidae) in northern Ukraine. Acta Parasitologica, 65, 341–353. 31974765 10.2478/s11686-019-00164-3

[45] Labbé A. 1894. Recherches zoologiques et biologiques sur les parasites endoglobulaires du sang des vertébrés. Archives de Zoologie Expérimentale et Générale, 2, 55–258.

[46] Labuda M. 1977. Mosquitoes (Diptera, Culicidae) in Záhorská nížina (west Slovakia). Entomologické Problémy, 14, 123–173 (in Slovak).

[47] Lankester ER. 1871. On *Undulina*, the type of a new group of infusoria. Journal of Cell Science, s2-11, 387–389.

[48] Lankester ER. 1882. On *Drepanidium ranarum*, the cell-parasite of the frog’s blood and spleen (Gaule’s wurmschen). Journal of Cell Science, s2-22, 53–65.

[49] Leveille AN, Ogedengbe ME, Hafeez MA, Tu HH, Barta JR. 2014. The complete mitochondrial genome sequence of *Hepatozoon catesbianae* (Apicomplexa: Coccidia: Adeleorina), a blood parasite of the green frog, *Lithobates* (formerly *Rana*) *clamitans*. Journal of Parasitology, 100, 651–656. 24820055 10.1645/13-449.1

[50] Léveillé AN, Zeldenrust EG, Barta JR. 2021. Multilocus genotyping of sympatric *Hepatozoon* species infecting the blood of Ontario ranid frogs reinforces species differentiation and identifies an unnamed *Hepatozoon* species. Journal of Parasitology, 107, 246–261. 33780973 10.1645/20-18

[51] Levine ND. 1971. Taxonomy of the piroplasms. Transactions of the American Microscopical Society, 90, 2–33.

[52] Lim A, Shearer FM, Sewalk K, Pigott DM, Clarke J, Ghouse A, Judge C, Kang H, Messina JP, Kraemer MUG, Gaythorpe KAM, de Souza WM, Nsoesie EO, Celone M, Faria N, Ryan SJ, Rabe IB, Rojas DP, Hay SI, Brownstein JS, Golding N, Brady OJ. 2025. The overlapping global distribution of dengue, chikungunya, Zika and yellow fever. Nature Communications, 16, 3418. 10.1038/s41467-025-58609-5 PMC11986131 40210848

[53] Lunghi E, Ficetola GF, Mulargia M, Cogoni R, Veith M, Corti C, Manenti R. 2018. *Batracobdella leeches*, environmental features and *Hydromantes salamanders*. International Journal for Parasitology: Parasites and Wildlife, 7, 48–53. 29988806 10.1016/j.ijppaw.2018.01.003 PMC6031966

[54] Malysheva MN. 2009. Fauna of haemoparasites of batrachians (Amphibia, Anura) in Kyrgyzstan. Parazitologiia, 43, 32–45 [in Russian]. 19370979

[55] Mann KH. 1955. The ecology of the British freshwater leeches. Journal of Animal Ecology, 24, 98–119.

[56] Manwell RD. 1964. The genus *Dactylosoma*. Journal of Protozoology, 11, 526–530. 14231179 10.1111/j.1550-7408.1964.tb01792.x

[57] McCallum ML, Trauth SE. 2007. Physiological trade-offs between immunity and reproduction in the northern cricket frog (*Acris crepitans*). Herpetologica, 63, 269–274.

[58] Melicherová J, Ilgová J, Kváč M, Sak B, Koudela B, Valigurová A. 2014. Life cycle of *Cryptosporidium muris* in two rodents with different responses to parasitization. Parasitology, 141, 287–303. 24128742 10.1017/S0031182013001637

[59] Merilä J, Sterner M. 2002. Medicinal leeches (*Hirudo medicinalis*) attacking and killing adult amphibians. Annales Zoologici Fennici, 39, 343–346.

[60] Miyata A. 1976. Anuran haemoprotozoa found in the vicinity of Nagasaki City. 2. *Dactylosoma* ranarum (Kruse, 1890). Tropical Medicine, 18, 135–141.

[61] Morin CW, Comrie AC. 2013. Regional and seasonal response of a West Nile virus vector to climate change*.* Proceedings of the National Academy of Sciences, 110, 15620–15625. 10.1073/pnas.1307135110 PMC3785720 24019459

[62] Negm-Eldin MM. 1998. Life cycle, host restriction and longevity of *Babesiosoma mariae* Hoare, 1930 (Apicomplexa: Dactylosomatidae). Deutsche tierärztliche Wochenschrift, 105, 367–374. 9818523

[63] Netherlands EC. 2019. Ecology, systematics and evolutionary biology of frog blood parasites in northern KwaZulu-Natal. PhD Thesis, North-West University, Potchefstroom Campus.

[64] Netherlands EC, Cook CA, Du Preez LH, Vanhove MPM, Brendonck L, Smit NJ. 2020. An overview of the Dactylosomatidae (Apicomplexa: Adeleorina: Dactylosomatidae), with the description of *Dactylosoma kermiti* n. sp. parasitising *Ptychadena anchietae* and *Sclerophrys gutturalis* from South Africa. International Journal for Parasitology: Parasites and Wildlife, 11, 246–260. 32195110 10.1016/j.ijppaw.2019.12.006 PMC7078462

[65] Netherlands EC, Cook CA, Kruger DJD, du Preez LH, Smit NJ. 2015. Biodiversity of frog haemoparasites from sub-tropical northern KwaZulu-Natal, South Africa. International Journal for Parasitology: Parasites and Wildlife, 4, 135–141. 25830113 10.1016/j.ijppaw.2015.01.003 PMC4356870

[66] Nigrelli RF. 1929. *Dactylosoma jahni* sp. nov., a sporozoan parasite of the erythrocytes and erythroplastids of the newt (*Triturus viridescens*). Journal of Parasitology, 16, 102.

[67] Nöller W. 1913. Die Blutprotozoen des Wasserfrosches und ihre Uebergang. Archiv für Protistenkunde, 31, 169–240.

[68] O'Donoghue P. 2017. Haemoprotozoa: Making biological sense of molecular phylogenies. International Journal for Parasitology: Parasites and Wildlife, 6, 241–256. 28913164 10.1016/j.ijppaw.2017.08.007 PMC5582378

[69] Országh I, Halgoš J, Jalili N, Labuda M. 2001. Mosquitoes (Diptera, Culicidae) of Slovakia II. European Mosquito Bulletin, 11, 1–26.

[70] Paterson WB, Desser SS, Barta JR. 1988. Ultrastructural features of the apical complex, pellicle, and membranes investing the gamonts of *Haemogregarina magna* (Apicomplexa: Adeleina). Journal of Protozoology, 35, 73–80. 3130480 10.1111/j.1550-7408.1988.tb04080.x

[71] Pavľáková B, Pipová N, Balogová M, Majláth I, Mikulíček M, Majláthová V. 2024. Blood parasites of water frogs (*Pelophylax esculentus* complex) from the Danube Delta, Romania. Parasitology International, 102, 102920. 38969332 10.1016/j.parint.2024.102920

[72] Peskova T, Bachevskaya O, Plotnikov G. 2018. Hemoparasites of the lake frog *Pelophylax ridibundus* (Pallas, 1771) (Ranidae, Anura) inhabiting reservoirs of the North-Western Ciscaucasia. Current Studies in Herpetology, 18, 146–152.

[73] Plötner J. 2005. Die westpaläarktischen Wasserfrösche. Von Märtyrern der Wissenschaft zur biologischen Sensation. Bielefeld: Laurenti Verlag.

[74] R Core Team. 2024. R: A language and environment for statistical computing. R Foundation for Statistical Computing, Vienna, Austria. https://www.R-project.org

[75] Raffel TR, Rohr JR, Kiesecker JM, Hudson PJ. 2006. Negative effects of changing temperature on amphibian immunity under field conditions. Functional Ecology, 20, 819–828.

[76] Rahman WA, Shakinah Z. 2015. Influence of some environmental parameters on some frog populations and their parasitc fauna. Journal of Veterinary Science & Technology, 6, 227.

[77] Rambaut A. 2018. FigTree version 1.4.4. Computer program and documentation distributed by the author. http://tree.bio.ed.ac.uk/software/figtree

[78] Readel AM, Goldberg TL. 2010. Blood parasites of frogs from an equatorial African montane forest in western Uganda. Journal of Parasitology, 96, 448–450. 19958047 10.1645/GE-2284.1

[79] Reinhold JM, Halbert E, Roark M, Smith SN, Stroh KM, Siler CD, McLeod DS, Lahondère C. 2023. The role of *Culex territans* mosquitoes in the transmission of *Batrachochytrium dendrobatidis* to amphibian hosts. Parasites & Vectors, 16, 424. 37974288 10.1186/s13071-023-05992-x PMC10655354

[80] Rivosecchi L, Addonisio M, Maiolini B. 2007. I Ditteri Simulidi. Nuove chiavi dicotomiche per l'identificazione delle specie italiane con brevi note bio-tassonomiche. Trento: Quaderni del Museo Tridentino di Scienze Naturali.

[81] Ronquist F, Teslenko M, van der Mark P, Ayres DL, Darling A, Höhna S, Larget B, Liu L, Suchard MA, Huelsenbeck JP. 2012. MrBayes 3.2: Efficient Bayesian phylogenetic inference and model choice across a large model space. Systematic Biology, 61, 539–542. 22357727 10.1093/sysbio/sys029 PMC3329765

[82] Ruiz A. 1959. Sobre la presencia de un *Dactylosoma* en *Bufo marinus*. Revista de Biología Tropical, 7, 113–117.

[83] Saucedo B, Hughes J., Spitzen-van der Sluijs A., Kruithof N., Schills M., Rijks JM, *et al.* 2018. Ranavirus genotypes in the Netherlands and their potential association with virulence in water frogs (*Pelophylax* spp.). Emerging Microbes & Infections, 7, 1–14. 29615625 10.1038/s41426-018-0058-5 PMC5882854

[84] Saunders DC. 1960. A survey of the blood parasites in the fishes of the Red Sea. Transactions of the American Microscopical Society, 79, 239–252. 5936509

[85] Seabra-Babo J., Maia JP, Harris DJ. 2015. Scanning for apicomplexan parasites (Suborder Adeleorina) in five Holarctic anuran species. Herpetozoa, 27, 168–172.

[86] Schmittner SM, McGhee RB. 1961. The intra-erythrocytic development of *Babesiosoma stableri* n. sp. in *Rana pipiens pipiens*. Journal of Protozoology, 8, 381–386.

[87] Scholtyseck E, Mehlhorn H, Friedhoff K. 1970. The fine structure of the conoid of sporozoa and related organisms. Zeitschrift für Parasitenkunde, 34, 68–94. 10.1007/BF00260383 4993041

[88] Schwetz J. 1930. Notes protozoologiques. Les hématozoaires des grenouilles et des crapauds de Stanleyville (Congo Belge). Annales de Parasitologie Humaine et Comparée, 8, 122–134.

[89] Simmaco M, Kreil G, Barra D. 2009. Bombinins, antimicrobial peptides from *Bombina* species. Biochimica et Biophysica Acta, 1788, 1551–1555. 19366600 10.1016/j.bbamem.2009.01.004

[90] Speybroeck J, Beukema W, Bok B, Van Der Voort J. 2021. Field guide to the amphibians and reptiles of Britain and Europe. London: Bloomsbury Publishing.

[91] Stamatakis A. 2014. RAxML version 8: a tool for phylogenetic analysis and post-analysis of large phylogenies. Bioinformatics, 30, 1312–1313. 24451623 10.1093/bioinformatics/btu033 PMC3998144

[92] Stehbens WE. 1966. The ultrastructure of *Lankesterella hylae*. Journal of Protozoology, 13, 63–73. 4958002 10.1111/j.1550-7408.1966.tb01871.x

[93] Strelková L, Halgoš J. 2012. Mosquitoes (Diptera, Culicidae) of the Morava River floodplain, Slovakia. Central European Journal of Biology, 7, 917–926.

[94] Takken W, Knols BGJ, Sutcliffe JF. 2023. Olfaction in vector-host interactions. Wageningen Academic.

[95] Tamura K., Stecher G., Kumar S. 2021. MEGA11: Molecular evolutionary genetics analysis version 11. Molecular Biology and Evolution, 38, 3022–3027. 33892491 10.1093/molbev/msab120 PMC8233496

[96] Tanabe M. 1931. Studies on the blood inhabiting Protozoa of the frog. Keijo Journal of Medicine, 2, 53–71.

[97] Tavaré S. 1986. Some probabilistic and statistical problems in the analysis of DNA sequences. Lectures on Mathematics in the Life Sciences, 17, 57–86.

[98] Tiberti R, Gentilli A. 2010. First report of freshwater leech *Helobdella stagnalis* (Rhyncobdellida: Glossiphoniidae) as a parasite of an anuran amphibian. Acta Herpetologica, 5, 255–258.

[99] Ujvari B, Madsen T, Olsson M. 2004. High prevalence of *Hepatozoon* spp. (Apicomplexa, Hepatozoidae) infection in water pythons (*Liasis fuscus*) from tropical Australia. Journal of Parasitology, 90, 670–672. 15270125 10.1645/GE-204R

[100] Úngari LP, Netherlands EC, Quagliatto Santos AL, de Alcantara EP, Emmerich E, da Silva RJ, O’Dwyer, L.H. 2020. A new species, *Dactylosoma piperis* n. sp. (Apicomplexa, Dactylosomatidae), from the pepper frog *Leptodactylus labyrinthicus* (Anura, Leptodactylidae) from Mato Grosso State, Brazil. Parasite, 27, 73. 33332263 10.1051/parasite/2020070 PMC7746082

[101] Úngari LP, Netherlands EC, Santos ALQ, de Alcantara EP, Emmerich E, da Silva RJ, O´Dwyer LH. 2022. Diversity of haemogregarine parasites infecting Brazilian anurans, with a description of new species of *Dactylosoma* (Apicomplexa: Adeleorina: Dactylosomatidae). Acta Parasitologica, 67, 1740–1755. 36264526 10.1007/s11686-022-00624-3

[102] Valigurová A, Koudela B. 2008. Morphological analysis of the cellular, interactions between the eugregarine *Gregarina garnhami* (Apicomplexa) and the epithelium of its host, the desert locust *Schistocerca gregaria*. European Journal of Protistology, 44, 197–207. 18304787 10.1016/j.ejop.2007.11.006

[103] Veith Y, Wende AL, Matuschewski K, Schaer J, Müller K, Bannert B. 2023. Molecular characterization of *Schellackia* parasites in an urban population of sand lizards (*Lacerta agilis*) from Berlin, Germany. Parasitology Research, 122, 1759–1764. 37222818 10.1007/s00436-023-07856-w PMC10348933

[104] Walton A. 1946. Protozoan parasites of the Bufoninae (Amphibia). Transactions of the Illinois State Academy of Science, 39, 143–147.

[105] Wenyon CM. 1926. Protozoology: a manual for medical men, veterinarians and zoologists. Baillière, Tindall and Cox.

[106] Werner JK. 1993. Blood parasites of amphibians from Sichuan Province, People's Republic of China. Journal of Parasitology, 79, 356–363. 8501591

[107] Wickham H, François R, Henry L, Müller K, Vaughan D. 2023. dplyr: A grammar of data manipulation. Available from: https://CRAN.R-project.org/package=dplyr

[108] Wickham H, Vaughan D, Girlich M. 2024. tidyr: Tidy messy data. Available from: https://CRAN.R-project.org/package=tidyr

[109] Young S, Warner J, Speare R, Berger L, Skerratt LF, Muller R. 2012. Hematologic and plasma biochemical reference intervals for health monitoring of wild Australian tree frogs. Veterinary Clinical Pathology, 41, 478–492. 23003118 10.1111/j.1939-165X.2012.00470.x

[110] Zechmeisterová K, Javanbakht H, Kvičerová J, Široký P. 2021. Against growing synonymy: Identification pitfalls of *Hepatozoon* and *Schellackia* demonstrated on North Iranian reptiles. European Journal of Protistology, 79, 125780. 34020115 10.1016/j.ejop.2021.125780

